# Cerebrospinal fluid cytokines after autologous haematopoietic stem cell transplantation and intrathecal rituximab treatment for multiple sclerosis

**DOI:** 10.1093/braincomms/fcad011

**Published:** 2023-01-20

**Authors:** Joachim Burman, Christina Zjukovskaja, Anders Svenningsson, Eva Freyhult, Anna Wiberg, Kim Kultima

**Affiliations:** Department of Medical Sciences, Neurology, Uppsala University, Uppsala SE-751 85, Sweden; Department of Medical Sciences, Neurology, Uppsala University, Uppsala SE-751 85, Sweden; Department of Clinical Sciences, Karolinska Institutet Danderyd Hospital, Stockholm SE-171 77, Sweden; Department of Cell and Molecular Biology, Uppsala University, Uppsala SE-751 23, Sweden; Department of Medical Sciences, Neurology, Uppsala University, Uppsala SE-751 85, Sweden; Department of Immunology, Genetics and Pathology, Uppsala University, Uppsala SE-751 85, Sweden; Department of Medical Sciences, Clinical Chemistry, Uppsala University, Uppsala SE-751 85, Sweden

**Keywords:** multiple sclerosis, cytokines, autologous haematopoietic stem cell transplantation, rituximab, CSF

## Abstract

Multiple sclerosis has been established as an inflammatory disease of the central nervous system. Many aspects of the pathophysiology are still unknown and it is presently unclear how different treatments affect the immunopathology of multiple sclerosis. In this study, we explored cytokines discriminating between individuals with multiple sclerosis and healthy controls and then how these cytokines were affected by treatment intervention with autologous haematopoietic stem cell transplantation or intrathecal rituximab. CSF from individuals with multiple sclerosis and healthy controls were analysed with a proximity extension assay to simultaneously determine the level of 92 cytokines and other inflammation-related proteins. In total, CSF from 158 multiple sclerosis patients and 53 healthy controls were analysed. Sixty-four patients with relapsing-remitting multiple sclerosis and 27 with progressive multiple sclerosis took part in a cross-sectional study and underwent lumbar puncture on a single occasion. Forty-five patients with relapsing-remitting multiple sclerosis were treated with autologous haematopoietic stem cell transplantation and underwent lumbar puncture at baseline and then at follow-up visits made at 1-, 2- and 5 years. Twenty-two patients with progressive multiple sclerosis were treated with intrathecal rituximab and followed with lumbar punctures at baseline and then at follow-up visits made at 3-, 6- and 12 months. Of the 92 studied cytokines, 16 were found to be altered in multiple sclerosis and 11 were decreased after treatment with autologous haematopoietic stem cell transplantation. None of the studied cytokines was affected by treatment with intrathecal rituximab for progressive multiple sclerosis. Some proteins were highly associated with each other. Therefore, a cluster analysis was made and then the highest-ranked protein from the four highest-ranked clusters was used for the subsequent analyses. CCL3, IL-12B, CXCL10 and IL-8 discriminated between multiple sclerosis patients and controls, but only IL-12B differed between patients with relapsing-remitting and progressive multiple sclerosis. The CSF concentrations of CCL3, IL-12B and CXCL10 were decreased after autologous haematopoietic stem cell transplantation, whereas IL-8 appeared to be unaffected by this intervention. High concentrations of IL-8 were associated with worse outcome in both treatment groups. Overall, the results suggest a profound effect of autologous haematopoietic stem cell transplantation on the inflammatory milieu of the CSF in multiple sclerosis.

## Teaser

Burman *et al.* measured CSF concentrations of 92 cytokines in patients with multiple sclerosis. CCL3, IL-12B, CXCL10 and IL-8 discriminated best between patients and healthy controls. The CSF concentrations of CCL3, IL-12B and CXCL10 decreased after treatment intervention with autologous haematopoietic stem cell transplantation for relapsing-remitting multiple sclerosis.

## Graphical Abstract

**Graphical abstract:**
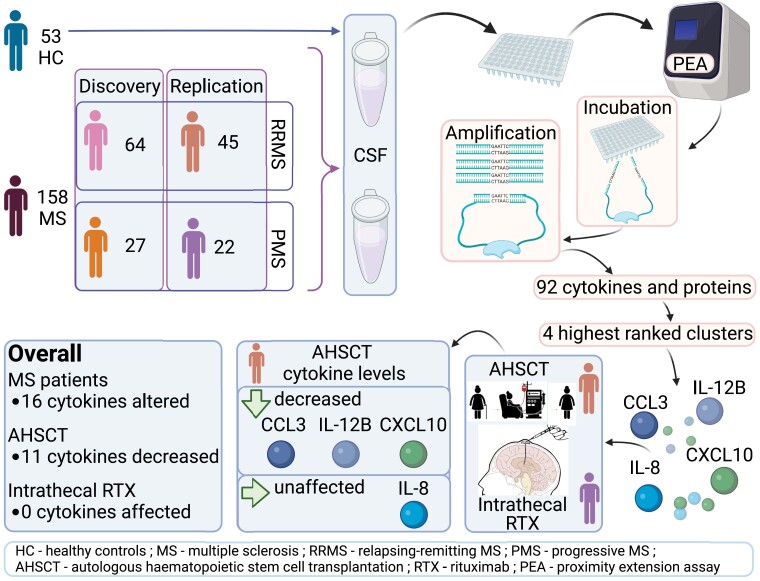

## Introduction

Multiple sclerosis has been firmly established as an inflammatory disease of the central nervous system. More than 200 risk genes have been identified, most of them associated with the immune system.^1^ Active multiple sclerosis plaques are hypercellular demyelinated lesions, containing infiltrates dominated by macrophages. Besides macrophages, inflammatory infiltrates are also composed of lymphocytes, the vast majority being cytotoxic T lymphocytes, with a lesser contribution from T helper cells, B cells and plasma cells.^2^ Several immune-based treatment strategies have successfully been implemented in health care, including interference with lymphocyte migration and selective lymphocyte depletion. Despite considerable effort, many details of the pathophysiology are still unknown and it is presently unclear how different treatments affect the complex immunopathology of multiple sclerosis.

Increasingly powerful technologies have made it possible to quantify a large number of proteins with great precision, which offers promise for the study of disease. One such technique is the proximity extension assay (PEA), where DNA oligonucleotide-labelled antibody pairs are extended by a DNA polymerase. The resulting DNA barcode can be amplified by polymerase chain reaction (PCR) and quantified by microfluidic qPCR. The PEA enables simultaneous analysis of up to 384 proteins in small volumes of biofluids. The assay requires two antibodies to be cross-linked in order to provide a signal, leading to reduced background noise and a high specificity, enabling simultaneous analysis of up to 384 proteins in small volumes of biofluids.^3^

In this study, we used PEA to quantify a panel of 92 proteins associated with inflammatory diseases and related biological processes in CSF from patients with multiple sclerosis. As a first aim, we determined which of these proteins were altered in patients with relapsing-remitting multiple sclerosis (RRMS) or progressive multiple sclerosis (PMS). We then posed the question, of whether any of these would be affected by therapeutic intervention. To this end, we used CSF from patients treated with autologous haematopoietic stem cell transplantation (AHSCT) for RRMS and intrathecal rituximab (ITRTX) for PMS.

## Materials and methods

### Ethical approval

The samples analysed in the present study came from three different sources. One was a cross-sectional study investigating CSF biomarkers in multiple sclerosis, approved by the Regional Ethics Review Board in Uppsala (Dnr 2008/182). Another was CSF from a study of patients treated with AHSCT for multiple sclerosis, approved by the Regional Ethics Review Board in Uppsala (2012/080/1). Finally, samples collected in the multicentre, prospective, open-label, phase 1b Intrathecal Treatment Trial in Progressive Multiple Sclerosis (ITT-PMS)^4^ approved by the Regional Ethics Review Board in Umeå (Dnr 2009-08-157 M). All participating subjects provided written informed consent.

### Subjects

The CSF concentration of inflammation-related proteins was determined by PEA in CSF samples from 158 multiple sclerosis patients and 53 healthy controls. Sixty-four RRMS and 27 PMS (23 secondary progressive multiple sclerosis (MS) and 4 primary progressive MS) patients took part in a cross-sectional study and underwent a single lumbar puncture. These patients constituted the discovery cohort. Forty-five RRMS patients were treated with AHSCT and underwent lumbar puncture at baseline and then 1-, 2- and 5 years after the therapeutic intervention. The final 22 patients were treated with ITRTX for PMS (14 secondary progressive MS and 8 primary progressive MS) and underwent lumbar puncture at baseline and then 3-, 6- and 12 months after the intervention. Baseline samples from the patients treated with AHSCT and ITRTX constituted the replication cohort. The controls were volunteers without neurological disease or other major diseases, such as cancer or systemic inflammatory disease. The patients from both patient cohorts were treated with various disease modifying drugs (DMD), which were grouped together to facilitate the statistical analysis. Dimethyl fumarate, glatiramer acetate, interferons, intravenous IgG and teriflunomide were considered first-line treatments. Fingolimod, mitoxantrone, natalizumab and rituximab were considered to be second-line treatments. The characteristics of patients and controls are summarized in Table 1.

**Table 1:** Clinical and demographical data

		Control	Discovery RRMS	Discovery PMS	AHSCT (baseline)	RTX (baseline)
*N*		(*N* = 53)	(*N* = 64)	(*N* = 27)	(*N* = 45)	(*N* = 22)
Sex	F/M	30/23	47/17	17/10	29/16	15/7
Age at inclusion	Mean (SD)	35 (15)	35 (9.8)	58 (8.3)	31 (6.5)	46 (7.9)
EDSS	Median [IQR]	*NA*	4.5 [3–6]	2 [1–3]	3.5 [2.5–4.0]	6.3 [5.25–6.5]
Disease duration (years)	Mean (SD)	*NA*	5.7 (7.7)	21 (11)	6.4 (5.6)	14 (7.6)
Number of previous treatments	Median [IQR]	*NA*	0 [0–1]	0 [0–1.5]	2 [1–3]	*NA*
All treatments	Naïve	*NA*	50	*NA*	13	*NA*
	First line^a^	*NA*	12	*NA*	11	*NA*
	Second line^b^	*NA*	2	*NA*	21	*NA*
NEDA/EDA					35/10	
CDW/stable						7/9^c^

Abbreviations: IQR = interquartile range; NA = not available

a First line: DMF = dimethyl fumarate; GLA = glatiramer acetate; IFN = interferon; IVIG = intravenous immune globulin; TF = teriflunomide.

b Second line: FLM = fingolimod; RTX = rituximab; MTX = mitoxantrone; NZB = natalizumab.

c For six patients, data were not available.

### Definitions of clinical events

A *clinical relapse* was defined as a period of acute worsening of neurological function lasting ≥24 h not attributable to an external cause such as increased body temperature or acute infection. An ‘MRI event’ was defined as the appearance of any T_2_ lesion >3 mm or gadolinium enhancing lesion in the brain or spinal cord not present on the baseline scan. The baseline scan was the last MRI scan made before treatment commenced. ‘Confirmed disability worsening (CDW)’ was defined as an increase in expanded disability status scale (EDSS) score with at least one point from baseline sustained between two follow-up visits separated in time by no less than 6 months (1.5 points if EDSS at baseline was 0 and 0.5 points if the baseline EDSS ≥ 5.5). ‘No evidence of disease activity-3 (NEDA-3)’ was defined as absence of clinical relapses, MRI events and CDW. Patients who did not maintain any NEDA-3 were considered to have ‘evidence of disease activity (EDA)’.

### Procedures

#### AHSCT

The details of the procedure have been described previously.^5^ In brief, haematopoietic stem cells were mobilized using a single dose of 2 g/m^2^ of cyclophosphamide followed by 5–10 µg/kg/day of filgrastim for 5 days. The non-myeloablative conditioning regimen consisted of cyclophosphamide (200 mg/kg) and anti-thymocyte globulin (6.0 mg/kg). DMDs were discontinued before the mobilization, with wash-out for natalizumab and fingolimod of at least 3 months.

#### Phase 1b intrathecal treatment trial in PMS

The details of the procedure have been described previously.^4^ In brief, a ventricular catheter was implanted into the right frontal horn and connected to a subcutaneous Ommaya reservoir. Patients were thereafter treated with three cycles of 25 mg rituximab (Mabthera^®^, Roche, Indianapolis, IN, USA) 1 week apart.

### PEA

CSF samples were sent in one batch to Olink Proteomics (Uppsala, Sweden) for quantification of 92 proteins included in the Olink Target 96 inflammation panel (Supplementary Table 1) using PEA. The Olink Target 96 inflammation panel includes a broad selection of proteins associated with biological functions linked to immune response and inflammatory diseases. The proteins were chosen by the manufacturer through an extensive process surveying public-access bioinformatic databases. In brief, this assay uses two separate antibodies binding to the same protein in the sample. Each antibody is coupled to one cDNA strand, which ligates when brought into proximity, is extended by a polymerase and is finally detected using a Biomark HD 96 × 96 dynamic real-time PCR arrays. Each sample is also spiked with two incubation controls: green fluorescent protein and phycoerythrin (one extension control and one detection control). These controls are used to determine the lower detection limit (negative control) and to normalize the measurements. A normalized protein expression (NPX) value for each protein in the sample is calculated by normalizing the CT values by subtracting the values for extension control, as well as an inter-plate control. The scale is shifted using a correction factor (normal background noise). The NPX values are in log_2_ scale. For details, see the service provider’s homepage (www.olink.com).

### Statistical analysis

The statistical analysis of the measured protein level was based on the NPX values in log_2_ scale. Linear regression and ANOVA *F*-tests were adopted to identify proteins that significantly differed between patient groups. Each protein was analysed separately, and the models were adjusted for age and sex by including them as covariates in the linear model. The *P*-values were adjusted for multiple tests using Benjamini–Hochberg’s FDR method. A protein was considered to differ significantly between the groups if the adjusted *P*-value (*q*-value) was ≤0.01. Linear mixed effects models analysis of deviance tests was adopted to identify proteins that differed significantly between time points; models were adjusted for age and sex as covariates, and patients were included as random effects variable. *Post hoc* analyses were performed using pairwise comparisons between groups using the estimated marginal means from a linear regression model or a linear mixed-effects model, using the R-package emmeans.^6^ A *post hoc* comparison was considered to differ significantly if the *P*-value was ≤0.05. The Pearson correlation coefficients between all proteins of interest were calculated and visualized in a heatmap. Further, hierarchical clustering was performed using average linkage and the correlation distance $\sqrt{1-r}$, where *r* is the correlation coefficient. The discriminative ability of a protein or a combination of proteins was assessed using logistic regression and evaluated using receiver operating characteristic curve (ROC) area under the curve (AUC). The AUC is reported together with a 95% bootstrap confidence interval computed using the R-function ci.roc in the pROC package.^7^ All statistical analyses and figures were computed in R-4.0.2.

## Results

### Sixteen proteins were differentially expressed in multiple sclerosis

**Figure 1:**
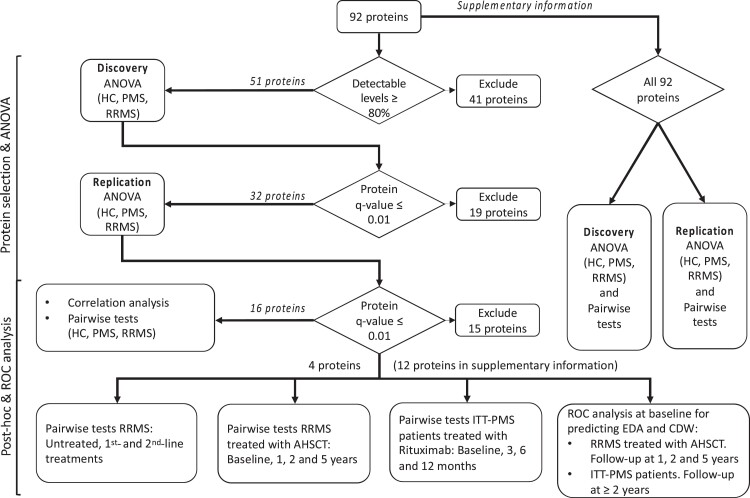
Overview of protein analysis and selection.

Fifty-one proteins had at least 80% values above lower level of detection across samples and were analysed further. In the discovery cohort, 32 proteins were found to differ significantly (*q-value* ≤ 0.01) between the groups (healthy controls, RRMS and PMS). Sixteen of these could be confirmed in the replication cohort (*q*-value ≤ 0.01) (Fig. 1): CCL3, IL-12B, sCD5, IL-8, CXCL10, CXCL11, CXCL1, CXCL9, TNFRSF9, TNFSF14, sCD8a, CCL4, MCP-2, CCL23, IL-18 and CCL19 (Table 2). Details on the complete analysis of all 92 proteins can be found in Supplementary Table 1.

**Table 2:** Sixteen proteins were significant different between Healthy Controls, PMS and RRMS in both discovery and replication cohort (ANOVA *q* < 0.01)

Protein	Discovery *P*-value	Discovery *q*-value	Replication *P*-value	Replication *q*-value
CCL3	1.7E-18	6.7E-17	2.4E-16	8.2E-15
IL-12B	2.6E-18	6.7E-17	2.0E-09	2.2E-08
CD5	2.4E-14	4.1E-13	6.0E-10	1.0E-08
IL8	1.5E-13	2.0E-12	8.3E-07	3.5E-06
CXCL10	2.8E-12	2.8E-11	6.1E-09	5.1E-08
CXCL11	1.8E-11	1.5E-10	8.0E-08	4.5E-07
CXCL1	5.1E-11	3.7E-10	2.1E-06	8.1E-06
CXCL9	9.1E-11	5.8E-10	1.5E-08	1.0E-07
TNFRSF9	3.6E-10	2.1E-09	1.4E-04	3.6E-04
TNFSF14	8.8E-08	4.5E-07	3.7E-07	1.8E-06
CD8A	1.4E-07	6.3E-07	8.8E-04	2.0E-03
CCL4	1.7E-07	7.3E-07	1.0E-04	2.9E-04
MCP-2	5.1E-07	2.0E-06	1.2E-05	4.2E-05
CCL23	8.8E-06	3.2E-05	6.3E-04	1.5E-03
IL18	4.1E-05	1.2E-04	3.3E-03	7.1E-03
CCL19	1.6E-04	3.9E-04	2.6E-05	7.9E-05

### Four clusters of correlating proteins could be identified

**Figure 2:**
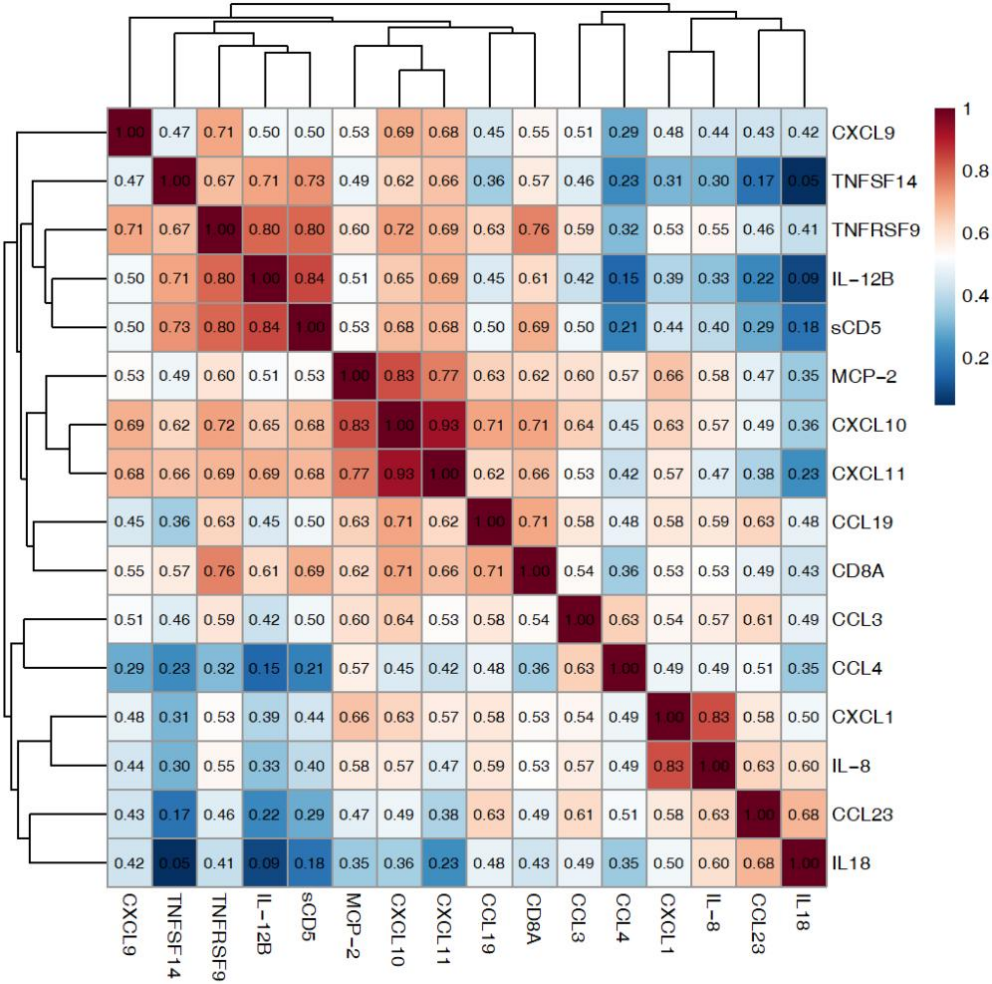
**Correlation matrix of the 16 proteins differentiating between MS patients and healthy controls.** Data from the exploration and replication cohort were combined and then the correlations between 16 proteins differentiating between MS patients and healthy controls were computed using Pearson correlation. Hierarchical clustering was performed using average linkage and the correlation distance $\sqrt{1-r}$, where *r* is the correlation coefficient. Three clusters of highly correlating (*r* > 0.75, *P* < 0.001) proteins could be identified. One cluster contained IL-12B, sCD5 and TNFRSF9, another CXCL10, CXCL11 and MCP-2, and the third cluster IL-8 and CXCL1. CCL3, with the lowest *P*-value in both the discovery and replication cohort, did not cluster with any other protein and was considered its own cluster.

The correlations between these 16 proteins based on baseline PMS and RRMS samples were computed and three clusters of highly correlating (*r* > 0.75, *P* < 0.001) proteins were found: (i) TNFRSF9, sCD5 and IL-12B; (ii) MCP-2, CXCL10 and CXCL11 and (iii) CXCL1 and IL-8. The remaining proteins did not cluster with any other proteins and were considered isolated clusters of single proteins. Among them was CCL3, with the lowest *P*-value in both the discovery and replication cohort (Fig. 2). The proteins in each cluster were ranked based on their *P*-values in the discovery cohort and the highest-ranked protein from the four highest-ranked clusters were used for the subsequent analyses. This narrowed down, but was not limited to, the analysis to four proteins of main interest, namely, CCL3, IL-12B, IL-8 and CXCL10.

### CCL3, IL-12B, IL-8 and CXCL10 were elevated in both PMS and RRMS patients

**Figure 3:**
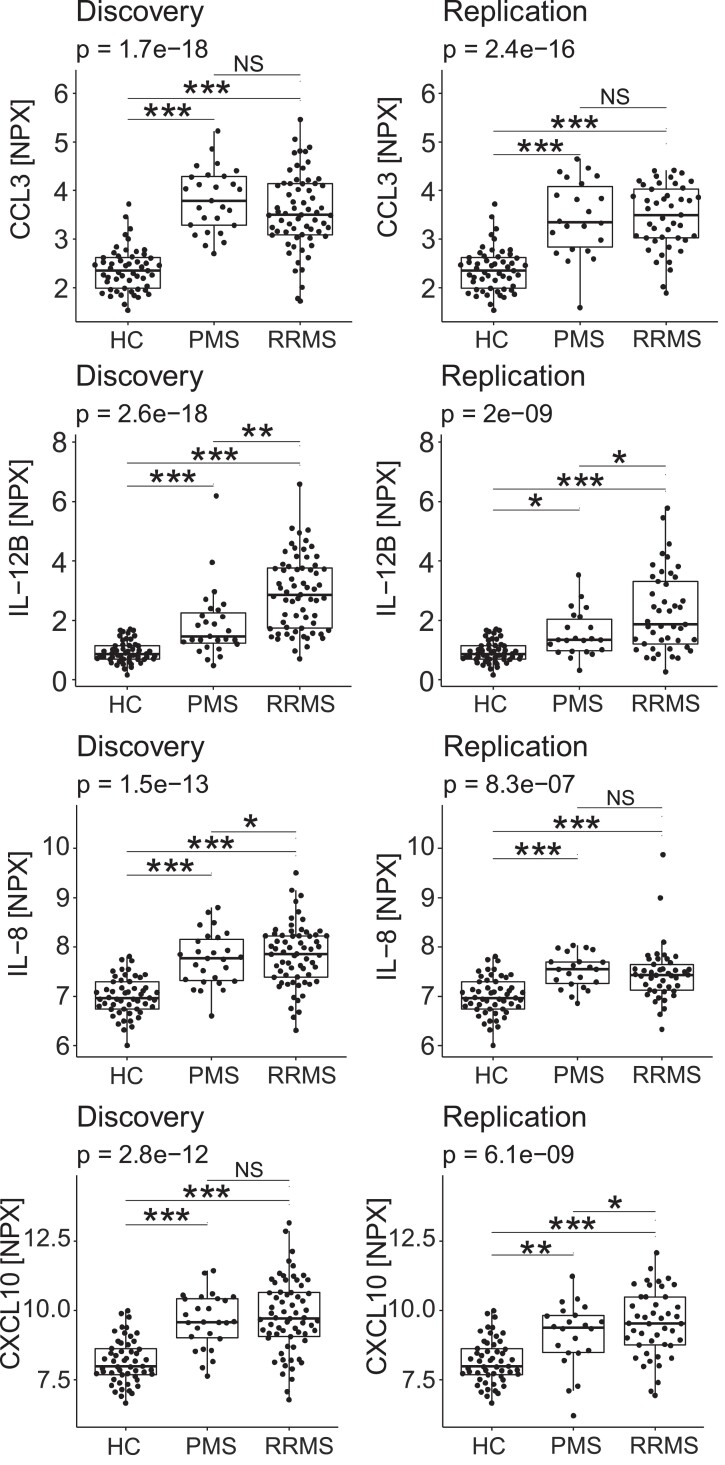
**CSF concentrations of CCL3, IL-12B, IL-8 and CXL10 in healthy controls, patients with relapsing-remitting MS and progressive MS.** Using linear models adjusted for sex and age, CCL3, IL-12B, IL-8 and CXCL10 were identified as the most important proteins differentiating between MS patients and healthy controls. Pairwise comparisons between groups using the estimated marginal means from the linear regression model showed that IL-12B was the only protein that was consistently higher in patients with RRMS than patients with PMS. Each datapoint represents a patient’s protein NPX value. NS = not significant, *P < 0.05, **P < 0.01, ***P < 0.001.

In both the discovery and replication cohort, all four proteins were increased in PMS patients in comparison to healthy controls (CCL3: *P*_dis_ < 0.001, *P*_rep_ < 0.001; IL-12B: *P*_dis_ < 0.001, *P*_rep_ < 0.05; IL-8: *P*_dis_ < 0.001, *P*_rep_ < 0.001 and CXCL10: *P*_dis_ < 0.001, *P*_rep_ < 0.01). Similarly, the four proteins were increased in RRMS patients in comparison to healthy controls (CCL3: *P*_dis_ < 0.001, *P*_rep_ < 0.001; IL-12B: *P*_dis_ < 0.001, *P*_rep_ < 0.001; IL-8: *P*_dis_ < 0.001, *P*_rep_ < 0.001 and CXCL10: *P*_dis_ < 0.001, *P*_rep_ < 0.001) (Fig. 3). Comparable results were observed for the correlating proteins (TNFRSF9, sCD5, MCP-2, CXCL11, CXCL1, all *P* < 0.001–*P* < 0.01), except for sCD5, MCP-2 and TNFRSF9, where the difference between PMS patients and healthy controls in the replication cohort was not significant (Supplementary Table 1 and Fig. 1).

### IL-12B was higher in RRMS patients than in patients with PMS

In both the discovery and replication cohort, IL-12B was higher in RRMS patients than in patients with PMS (*P*_dis_ < 0.01, *P*_rep_ < 0.05) (Fig. 3). The same observation was made for the correlating sCD5 (*P*_dis_ < 0.05, *P*_rep_ < 0.01) (Supplementary Table 1 and Fig. 1). IL-8 was higher in RRMS than PMS patients in the discovery cohort only (*P*_dis_ < 0.05) and CXCL10 was higher in RRMS than PMS patients in the replication cohort only (*P*_rep_ < 0.05) (Fig. 3).

### CCL3, IL-12B and CXCL10 were elevated in patients with first-line treatment

**Figure 4:**
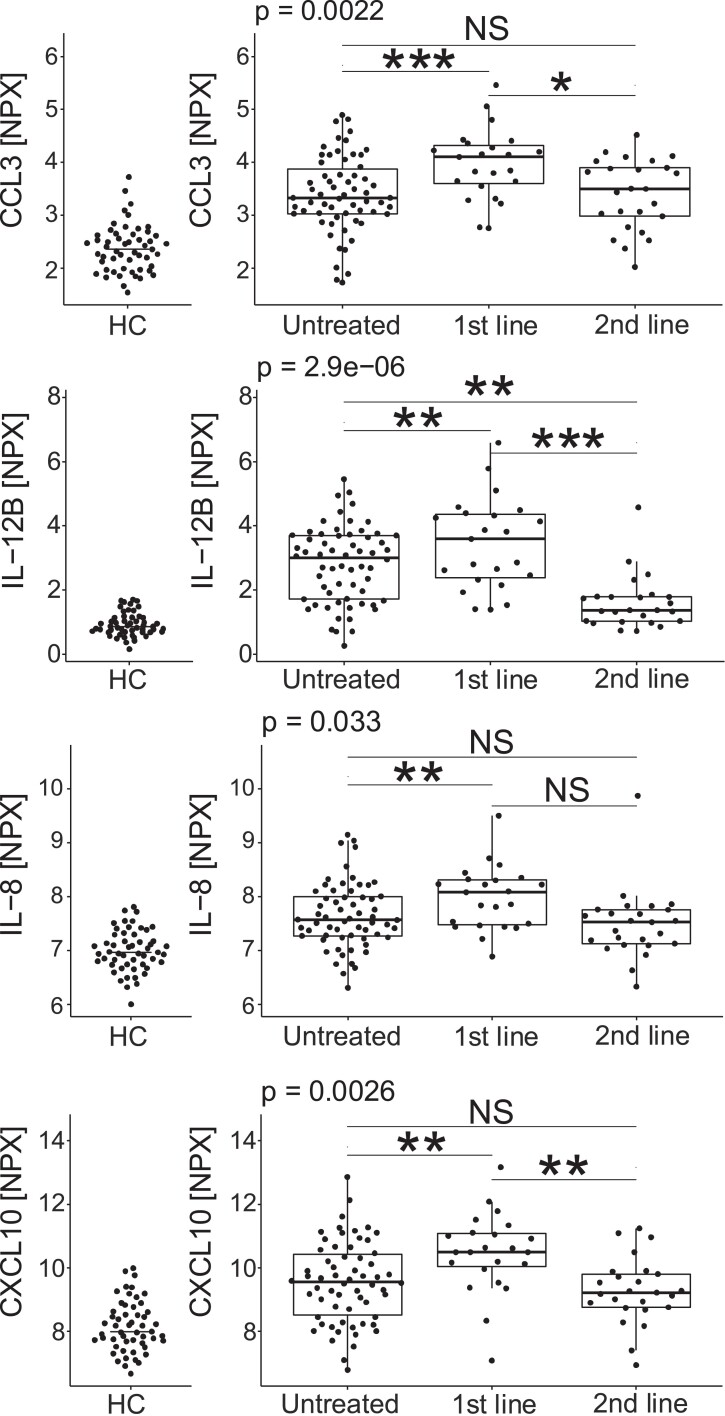
**CSF concentrations of CCL3, IL-12B, IL-8 and CXL10 in healthy controls, untreated patients and relapsing-remitting MS patients treated with first- and second-line treatment.** Treatment effects on baseline CCL3, IL-12B, IL-8 and CXCL10 were estimated using linear regression models adjusted for age and sex. Pairwise comparisons between treatments were estimated using the marginal means from the linear regression model. In general, patients with first-line treatment had higher concentrations than untreated patients and patients treated with second-line treatment. Each datapoint represents a patient’s protein NPX value. NS = not significant, *P < 0.05, **P < 0.01, ***P < 0.001.

To assess the impact of previous treatment, the RRMS patients of the discovery and replication cohort were pooled together and then separated by treatment status at baseline: untreated, treated with first- or second-line DMDs (Fig. 4). The CCL3 concentrations were increased in patients with ongoing first-line DMD treatment in comparison to untreated patients (*P* < 0.001) and patients treated with second-line DMDs (*P* < 0.05). The concentrations of IL-12B were also increased in patients with ongoing first-line DMD treatment in comparison to untreated patients (*P* < 0.01) and patients treated with second-line DMDs (*P* < 0.001). The second-line DMDs significantly suppressed the concentrations of IL-12B below the levels of untreated (*P* < 0.01). Similar results were seen with the correlating proteins, TNFRSF9 and sCD5, with significantly elevated concentrations in patients with ongoing 1st line DMD treatment compared to both patients with second-line DMD treatment (TNFRSF9: *P* < 0.001; sCD5: *P* < 0.001) and untreated (TNFRSF9: *P* < 0.01; sCD5: *P* < 0.05). The concentrations of sCD5 were also lower in the patients receiving second-line treatment in comparison to untreated patients (*P* < 0.001) (Supplementary Fig. 2). Patients treated with first-line DMDs had higher concentrations of IL-8 than untreated patients (*P* < 0.01), but the difference vis-à-vis patients treated with second-line DMDs was not statistically significant. Finally, the concentrations of CXCL10 were also increased in patients with ongoing first-line DMD treatment in comparison to untreated patients (*P* < 0.01) and patients treated with second-line DMDs (*P* < 0.01). Similar findings were made for the correlating CXCL11 (both *P* < 0.01), but for MCP-2 only the difference between first line and untreated was significant (*P* < 0.05). A full account of the results for all proteins is made in Supplementary Fig. 2 and Table 2.

### The concentrations of CCL3, IL-12B and CXCL10 were decreased at follow-up 1-, 2- and 5 years after AHSCT

**Figure 5:**
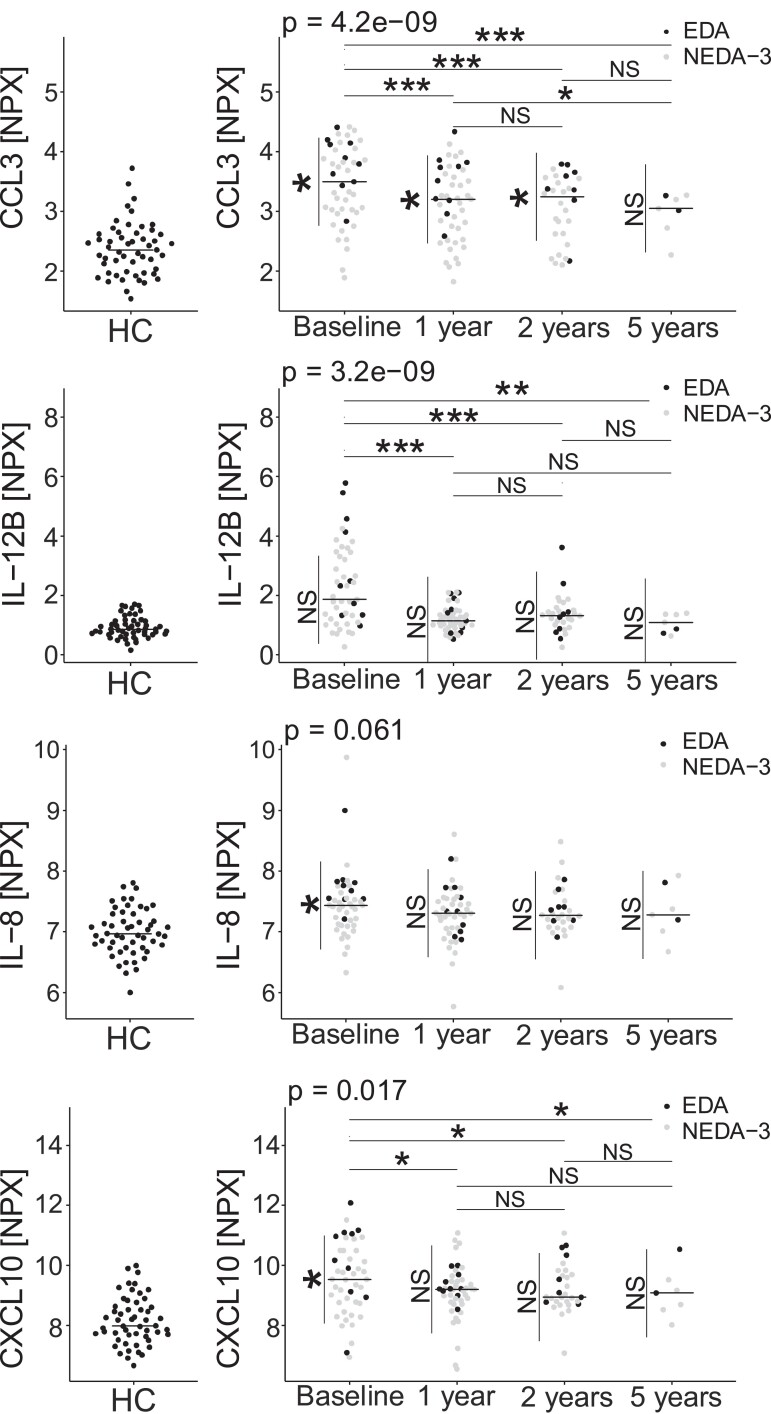
**CSF concentrations of CCL3, IL-12B, IL-8 and CXL10 in healthy controls and relapsing-remitting MS patients treated with AHSCT.** Linear mixed effects models adjusted for sex and age were used to estimate difference over time for the proteins CCL3, IL-12B, IL-8 and CXCL10. Pairwise comparisons between time points were estimated using the marginal means from the linear mixed linear models. After treatment with AHSCT, the levels of IL-12B decreased rapidly to approximately the level of healthy controls. CCL3 and CXCL10 also decreased, but more gradually and did not reach the levels of healthy controls. The levels of IL-8 were unaffected by AHSCT. The association with EDA was estimated within each time point using the marginal means from the model. Patients with evidence of EDA during follow-up had higher levels of CCL3, IL-8 and CXCL10. Each datapoint represents a patient’s protein NPX value at the given time point. NS = not significant, *P < 0.05, **P < 0.01, ***P < 0.001.

The protein concentrations of patients treated with AHSCT are shown in Fig. 5. The concentrations of CCL3 (*P*_1-year_ < 0.001), IL-12B (*P*_1-year_ < 0.001) and CXCL10 (*P*_1-year_ < 0.05) had decreased at follow-up 1 year after treatment compared with the baseline values, and remained decreased at the 2- and 5-years follow-ups (CCL3: *P*_2-years_ < 0.001, *P*_5-years_ < 0.001; IL-12B: *P*_2-years_ < 0.001, *P*_5-years_ < 0.01 and CXCL10: *P*_2-years_ < 0.05, *P*_5-years_ < 0.05). The concentration of CCL3 continued to decrease over the follow-up period and there was a decrease in the concentrations between the follow-up at 1- and 5 years after the intervention (*P* < 0.05). The correlating proteins TNFRSF9 and sCD5 showed similar results to IL-12B; both decreased in concentrations at follow-up at one (both *P* < 0.001), two (TNFRSF9 *P* < 0.001; sCD5 *P* < 0.05) and for TNFRSF9, also 5 years after the intervention (*P* < 0.05). MCP-2 also showed decreased concentrations, at follow-up 2- (*P* < 0.05) and 5 (*P* < 0.05) years after the intervention. In contrast, the concentrations of CXCL11 were stable after intervention. For IL-8, There was no statistically significant decrease in IL-8 (*P*_ANOVA_ = 0.061), but the correlating CXCL1 decreased significantly (*P*_1-year_ < 0.001, *P*_2-years_ < 0.01 and *P*_5-years_ < 0.05). A full account of the results for all proteins is provided in Supplementary Fig. 3 and Table 3.

### CCL3, IL-8 and CXCL10 were elevated in patients treated with AHSCT and evidence of disease activity during follow-up

Patients with EDA during follow-up after AHSCT had higher concentrations of CCL3, IL-8 and CXCL10 at baseline (all *P* < 0.05), in comparison to patients with NEDA (Fig. 5). Similar results were seen for the correlating proteins CD5, TNFRSF9, MCP-2 (all *P* < 0.05), CXCL1 (*P* < 0.01), as well as CXCL9 and CCL23 (both *P* < 0.05). The concentrations of CCL3 and CXCL1 were also elevated at follow-up after 1 year (both *P* < 0.05) and for CCL3 also at the 2-year follow-up (*P* < 0.05) (Supplementary Fig. 3 and Table 3).

### Treatment with ITRTX for PMS did not affect the protein levels

**Figure 6:**
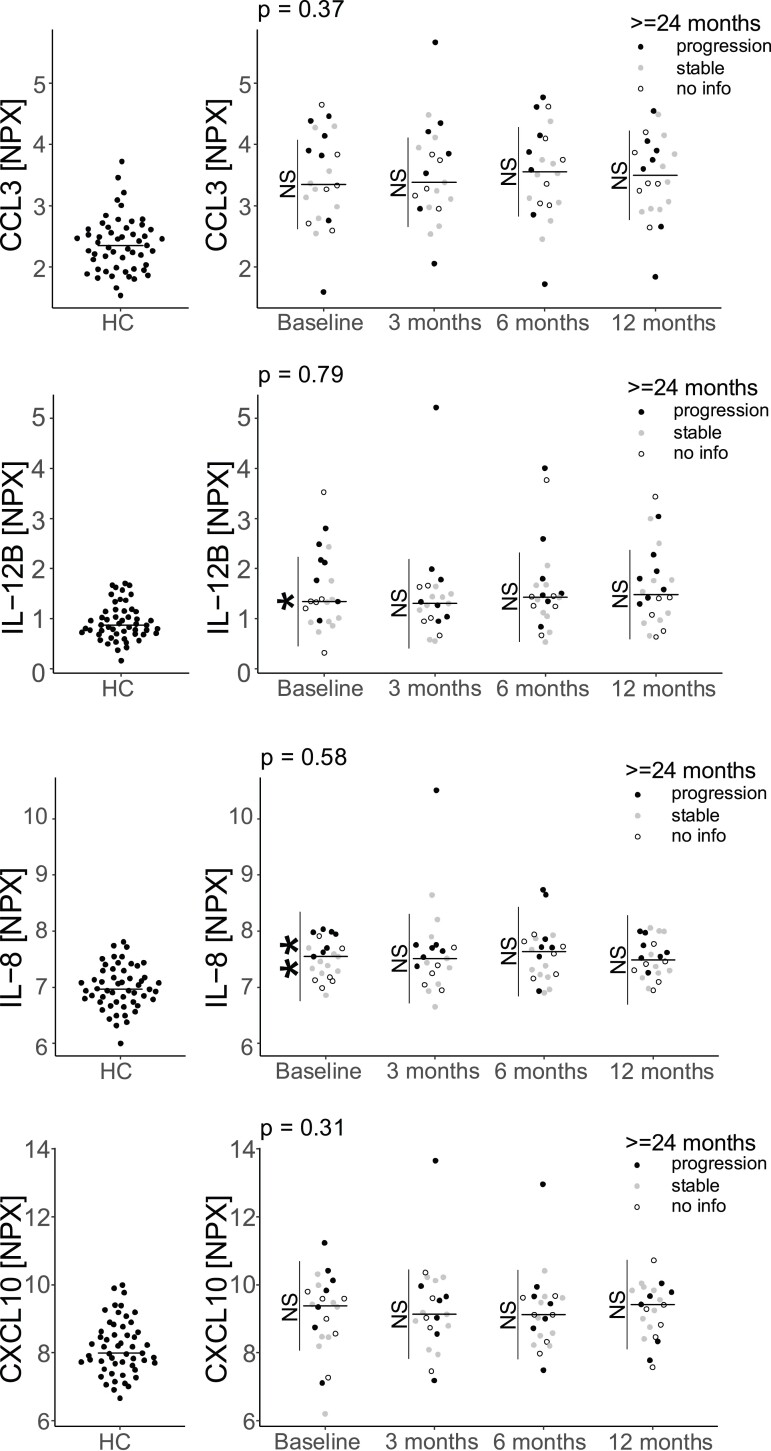
**CSF concentrations of CCL3, IL-12B, IL-8 and CXL10 in healthy controls and progressive MS patients treated with ITRTX.** Linear mixed effects models adjusted for sex and age were used to estimate the difference over time for the proteins CCL3, IL-12B, IL-8 and CXCL10. The levels of these cytokines were unaffected by treatment with ITRTX. The association with disability progression was estimated within each time point using the marginal means from the model. Patients with confirmed disability progression had higher levels of IL-12B and IL-8 at baseline. Each datapoint represents a patient’s protein NPX value at the given time point. NS = not significant, *P < 0.05, **P < 0.01.

The effect of intrathecally administered rituximab was evaluated at follow-up visits made at 3 months, 6 months and 12 months. There were no statistically significant differences in the concentrations of CCL3, IL-12B, IL-8 or CXCL10 between any of these time points (Fig. 6). We extended our analysis to all the 16 proteins that differed significantly but failed to detect any statistical significant changes, with the exception of an increase in CCL19 (*P*_6 months_ < 0.05, *P*_12 months_ < 0.001) (Supplementary Fig. 4). CDW was evaluated at last follow-up (≥24 months after intervention). Patients who experienced CDW had higher concentrations of IL-8 (*P* < 0.01), CXCL1 (*P* < 0.05) and IL-12B (*P* < 0.05) at baseline, in comparison to patients who were stable in EDSS during follow-up (Fig. 6, Supplementary Fig. 4 and Table 4).

### Baseline concentrations of IL-8, CXCL1 and TNSFR9 were strong predictors for subsequent disease activity

The concentrations of IL-8 and the correlating CXCL1 were significantly increased at baseline in RRMS patients experiencing EDA after AHSCT as well as in PMS patients treated with ITRTX who experienced CDW, suggesting that high concentrations of IL-8 may be associated with poor outcome. In a ROC analysis, IL-8 concentration at baseline was the best discriminator in both AHSCT-treated patients with EDA [AUC = 0.84 (95% confidence interval (CI): 0.69–0.95)] and PMS patients treated with ITRTX and CDW [AUC = 0.92 (95% CI: 0.80–1.0)]. CXCL1 [AUC_AHSCT_ = 0.77 (95% CI: 0.60–0.91) and AUC_ITRTX_ = 0.86 (95% CI: 0.61–1.0)] and TNSFR9 also displayed high AUC in both cohorts [AUC_AHSCT_ = 0.71 (95% CI: 0.51–0.87) and AUC_ITRTX_ = 0.69 (95% CI: 0.42–0.92)]. A full account of the results for all proteins is made in Supplementary Fig. 5 for RRMS patients and Supplementary Fig. 6 for PMS patients.

### IL-8 and CCL3 improved the identification of individuals with CDW in PMS patients treated with ITRTX

To investigate if the combination of measurements of two or more cytokines could improve the possibility to predict outcome, logistic models were trained and AUC was estimated using all possible combinations of IL-12B, CCL3, IL-8 and CXCL10. The combination of CCL3 and IL-8 was slightly better for predicting confirmed disability worsening in patients treated with ITRTX (AUC = 0.97, 0.85–1.0). Otherwise, no improvements could be demonstrated (Supplementary Fig. 7).

## Discussion

In this study, we used a highly sensitive assay containing a large panel of inflammation-related proteins. Through sophisticated analyses, we identified four cytokines with limited association with each other, that discriminated between patients with multiple sclerosis and healthy controls, namely, CCL3, IL-12B, CXCL10 and IL-8. We proceeded by investigating if these would be affected by therapeutic intervention with AHSCT for RRMS or ITRTX for PMS. The CSF concentrations of CCL3, IL-12B and CXCL10 were decreased after AHSCT, whereas the concentrations of IL-8 were unaffected. None of the cytokines was altered by treatment with ITRTX. Finally, we determined if any of the proteins were associated with a worse outcome. Increased levels of IL-8 at baseline were associated with EDA post-AHSCT, as well as progression in EDSS in PMS patients treated with ITRTX.

Many previous studies have investigated the levels of cytokines in CSF from multiple sclerosis patients and several promising biomarker candidates have been put forward. A recent meta-analysis extracted data from 226 studies encompassing 13 526 multiple sclerosis patients and 8428 controls showed that 13 CSF cytokines and 21 blood cytokines were significantly increased in multiple sclerosis patients in comparison to the controls, suggesting that cytokines have the potential to be used as biomarkers for multiple sclerosis.^8^ However, the results of these studies are difficult to compare since the patient cohorts were considerably heterogenous and different methods to quantitate the proteins in question were used. A significant shortcoming is that the relative importance of the studied cytokines has not been ascertained. Furthermore, the response to therapeutic interventions has been studied to a much lesser degree, presumably by the inconvenience of performing repeated lumbar punctures.

To overcome these shortcomings, we used a PEA panel of 92 cytokines and other inflammation-related proteins. We then performed a cluster analysis to determine how these cytokines correlated with each other and determined that the measurements of CCL3, IL-12B, CXCL10 and IL-8 contained the most information. We proceeded with an investigation of how these cytokines would respond to therapeutic intervention. We decided to investigate AHSCT for RRMS and intrathecal treatment with rituximab for PMS. Arguably, AHSCT is the most intense immune suppressive treatment and has demonstrated superior results to standard DMDs in a phase III clinical trial.^9^ Similarly, anti-CD20 therapy has been the most promising approach for progressive forms of multiple sclerosis^10^ and the intrathecal administration route may offer better penetrance of the drug into the CNS. The CSF concentrations of IL-12B and related cytokines decreased promptly after treatment with AHSCT, whereas the levels of CCL3 and CXCL10 declined more gradually and IL-8 was unaffected. Somewhat surprisingly, none of these cytokines was altered by treatment with ITRTX for PMS.

A similar approach was used in a recently published study^11^ and overall, our results were in concordance with each other. In total, they could reliably detect 45/92 proteins from the inflammation panel in the CSF of multiple sclerosis patients. Their selection process was a little bit different and they also included proteins with lower call rates in their analysis. Nevertheless, we could confirm their top eight proteins discriminating between multiple sclerosis patients and controls. Similar to this study, IL-12B, CD5 and CCL3 were identified as the three most important proteins to discriminate samples from people with multiple sclerosis and healthy controls.^11^ One notable difference is that IL-8 could not be confirmed to be higher in multiple sclerosis in one of the two investigated cohorts in this study. Another important difference is that they did not systematically study the effect of therapeutic intervention with AHSCT or ITRTX.

CCL3 (also known as macrophage inflammatory protein 1-α, MIP-1-α) is a chemokine that attracts eosinophils, monocytes, immature dendritic cells, B cells and CD8^+^ T cells.^12^ It has been reported that CCL3 is produced by microglia in patients with active multiple sclerosis^13,14^ and that T cells expressing CCR5 (the receptor for CCL3) are increased in frequency in the blood of multiple sclerosis patients.^13^ CCL3 was reported to be increased in CSF from multiple sclerosis patients for the first time more than 25 years ago,^15^ and this finding has later been confirmed in larger studies.^11,16^ CCL3 decreased moderately and slowly over the course of 5 years after AHSCT. Hypothetically, it may represent fading out of smouldering multiple sclerosis lesions in the absence of influx of inflammatory cells from the periphery.

IL-12B is a subunit shared by two related cytokines, IL-12 and IL-23. It has a molecular weight of 40 kDa and it is sometimes also referred to as IL-12p40. IL-12B is secreted in excess over the other subunits of IL-12 and IL-23 and can exist in a monomeric or homodimeric form. It is primarily produced by activated immune cells including macrophages, neutrophils, microglia and dendritic cells.^17^ Mono/dimeric IL-12B is believed to have a function independent of the IL-12 and IL-23 receptor and is a chemoattractant for macrophages, which induces dendritic cell migration and the production of IFN-γ in CD8^+^ T cells.^18^ A previous study in multiple sclerosis patients showed that IL-12B mRNA from peripheral blood mononuclear cells increased preceding clinical relapse.^19^ IL-12B mRNA has also been found in multiple sclerosis lesions from autopsied patients.^20^ It has been reported that IL-12B, but not IL-12 or IL-23, was increased in CSF from multiple sclerosis patients^21,22^ and that the source of the increased IL-12B was intrathecal.^22^ IL-12B correlates with other measures such as CSF white blood cell count, IgG index and gadolinium enhancing lesions and most likely represents acute inflammation.^21^ In this study, we could confirm earlier reports that IL-12B was increased in multiple sclerosis.^11,21,22^ The levels of IL-12B were higher in RRMS patients than in patients with PMS, but both patients groups had clearly higher levels than healthy controls. The production of IL-12B was quickly normalized in all patients treated with AHSCT, the exception being some of the few patients displaying disease activity post-AHSCT. The concentrations of IL-12B clustered with soluble CD5, TNFRSF9, TNFSF14 and CXCL9, suggesting that they are part of the same pathway. All of these proteins significantly and rapidly decreased after treatment with AHSCT, but soluble CD5 and TNFRSF9 correlated best with IL-12B. CD5 is a lymphoid-specific transmembrane glycoprotein constitutively expressed on thymocytes and mature T and B1a lymphocytes. Current data support the view that membrane-bound CD5 is a negative regulator of antigen-specific receptor-mediated signalling in these cells, although the ligand of CD5 remains unidentified. A soluble form of CD5 comprises the three ectodomains and is generated after T-cell receptor activation by proteolytic excision of membrane-bound CD5.^23^ Overexpression of soluble CD5 leads to more severe forms of disease in animal models of arthritis and neuroinflammation, suggesting a role in autoimmune disease.^24^ TNFRSF9 (also known as CD137) is a potent costimulatory receptor and several agonistic anti-TNFRSF9 antibodies are currently in clinical trials for tumour immunotherapy. Paradoxically, TNFRSF9 agonists have also been reported to ameliorate autoimmune encephalomyelitis^25^ and other animal models of autoimmunity.^26^ Soluble forms of TNFRSF9 antagonise membrane-bound TNFRSF9 and therapeutic TNFRSF9 agonists. Increased levels of soluble TNFRSF9 have been reported in sera from patients with autoimmune diseases, suggesting that it may act as a natural regulator of immune responses.^26^

CXCL10 (also known as interferon gamma-induced protein 10, IP-10) is a well-characterized chemokine that attracts Th1 cells. As the older name implies, it is produced in macrophages, in response to IFN-γ. It has also many other functions, including inhibition of cytokine-stimulated haematopoietic progenitor cell proliferation and angiogenesis.^12^ In the CNS CXCL10 is produced by astrocytes and microglia.^27,28^ In post-mortem tissue from diseased multiple sclerosis patients, it has been demonstrated that CXCL10 was expressed by macrophages within the plaque and by reactive astrocytes in the surrounding parenchyma and the ligand CXCR3 was expressed by T cells and by astrocytes within the plaque.^29^ Several studies have found CXCL10 to be increased in CSF from multiple sclerosis patients,^30^ with the highest levels seen in patients with active disease.^31^ It has also been reported that CXCL10 decreases after initiation of natalizumab^32^ and rituximab^33^ treatment, but not to the level of healthy controls. We observed a similar modest decrease in CXCL-10 after AHSCT, but in similarity to CCL3, the levels of CXCL10 were still high in comparison to the levels of healthy controls up to 5 years after AHSCT. The reason for this is unclear, but may be an effect of lingering astrocyte and microglia activation.

IL-8 (also known as CXCL8) is a chemokine secreted by a wide variety of cells including astrocytes, microglia, endothelial cells and immune cells in response to a wide variety of natural and artificial stimuli. Significantly increased levels of IL-8 have been observed in a variety of inflammatory and autoimmune conditions, but it is also important for trafficking of leukocytes to the CNS.^21,34,35^ In difference to IL-12B, IL-8 does not seem to reflect acute inflammation in the CNS.^21^ IL-8 concentration was increased in the CSF from multiple sclerosis patients in several studies,^21,36,37^ and has also been linked to a worse prognosis. Higher baseline IL-8 was associated with conversion from radiologically and clinically isolated syndrome to definitive multiple sclerosis in one study,^37^ and subsequent relapses and gadolinium enhancing lesions in another study.^36^ It has also been linked to worse prognosis in traumatic brain injury.^38^ IL-8 is tightly connected to CXCL1 and in this study, both were increased in both RRMS and PMS to a similar degree. IL-8 was not affected by treatment with AHSCT or ITRTX, suggesting that it is not necessarily associated with inflammation in multiple sclerosis *per se*, but rather a consequence of previous damage to the CNS. High baseline levels of IL-8 were also associated with active disease in RRMS patients post-AHSCT and progression of EDSS in patients treated with ITRTX for PMS, which fits the concept that IL-8 act as a danger signal in the CNS.

As a side finding, it was noted that patient treated with first-line DMDs had higher levels than patients treated with second-line DMDs in 11 of the 16 cytokines altered in multiple sclerosis. This suggests that first-line DMDs are not able to supress inflammation to a sufficient degree. A majority of the patients treated with AHSCT had received second-line treatment with drugs such as natalizumab and rituximab before AHSCT. Most likely, the cytokine levels were affected by this and this may have led to an underestimation of the treatment effect of AHSCT. Taking this into account, it is possible that more than the 11 cytokines that we identified could be affected by immunosuppressive treatment. Nevertheless, it is clear that many of the cytokines were not normalized by AHSCT despite excellent clinical outcome with maintenance of no evidence of disease activity in a majority of patients. The reason for this is unclear, but it may represent danger signals emitted from damaged tissue as discussed above. Overall, patients with PMS had lower levels of IL-12B and its related cytokines, suggesting that acute inflammation is less prevalent in this course of multiple sclerosis. The cytokine levels in patients with PMS were largely unaffected by treatment with ITRTX, reinforcing that increased levels of the astrocyte and glia-associated chemokines are less connected to inflammation and more linked to glial biology. It also strongly suggests that immunosuppression is not the way forward to find effective therapies for PMS.

The PEA is a very sensitive method to detect proteins at low concentrations. The lower limits of quantification have been determined to be <1 pg/µL for 43/92 proteins and 1–10 pg/µL for 28/92 proteins in the inflammation panel, which is better than most other analysis options. Furthermore, the assay requires two antibodies to be cross-linked in order to provide a signal, which leads to reduced background and a high degree of specificity. We could reliably detect 51/92 proteins from the inflammation panel. Among the cytokines that could not be detected were IFN-γ, IL-17A and tumor necrosis factor (TNF)-α, which have been linked to the pathophysiology of multiple sclerosis. The most likely explanation for this is that such cytokines act locally and have a rapid turn-over so that they do not reach the CSF to any significant degree. In support of this view, we have previously demonstrated that there is a poor correlation between cytokines that can be detected in tissue and cytokines that can be found in the CSF.^39^

The PEA panels are fixed to a prespecified set of proteins, relevant to different areas of interest. We used a panel containing 92 proteins relevant to inflammatory processes. The main advantage of this approach was that we were able to simultaneously measure a large number of proteins in a previously validated set-up. Nevertheless, the fixed set of proteins in the inflammation panel was also a limitation of this study. Some cytokines that were previously identified as biomarkers of multiple sclerosis were not contained in the panel. Most notably CXCL13, which has been the subject of numerous studies in the past and is related to disease activity.^21,40^ CXCL8, CXCL12, CXCL13, CCL5, CCL21, CCL22 and IL-15 have also been reported to be differentially expressed in multiple sclerosis,^8,41^ but were not included in the inflammation panel. Another limitation is that the PEA currently is a semi-quantitative method that does not yield absolute values in the concentrations of the measured proteins.

The PEA enabled us to study the CSF of multiple sclerosis patients with a high degree of granularity, which hitherto had not been possible. We could reliably detect 51 proteins, 16 of them were altered in multiple sclerosis, suggesting that they are relevant for the pathophysiology of multiple sclerosis. With some exceptions, these proteins were equally increased in patients with RRMS and PMS, suggesting that the inflammatory environment is quite similar between these disease courses. A majority of the altered proteins were decreased after treatment intervention with AHSCT, reflecting the good clinical outcome seen in most patients treated with AHSCT for RRMS. In contrast, we could not detect any effect of ITRTX for PMS, highlighting the need for alternative treatment approaches that target something else than inflammation in patients with PMS.

## Supplementary Material

fcad011_Supplementary_Data

## Data Availability

The data that support the findings of this study are available from the corresponding author, upon reasonable request.

## Abbreviations

AHSCT =: autologous haematopoietic stem cell transplantation
AUC =: area under the curve
CDW =: confirmed disability worsening
CI =: confidence interval
DMD =: disease modifying drugs
DMF =: dimethyl fumarate
EDA =: evidence of disease activity
EDSS =: expanded disability status scale
FDR =: false discovery rate
FLM =: fingolimod
GLA =: glatiramer acetate
HC =: healthy controls
IFN =: interferon
IQR =: interquartile range
IVIG =: intravenous immune globulin
ITRTX =: intrathecal rituximab
MS =: multiple sclerosis
MTX =: mitoxantrone
NA =: not available
NEDA-3 =: no evidence of disease activity-3
NPX =: normalized protein expression
NZB =: natalizumab
PCR =: polymerase chain reaction
PEA =: proximity extension assay
PMS =: progressive multiple sclerosis
RRMS =: relapsing-remitting multiple sclerosis
RTX =: rituximab
ROC =: receiver operating characteristic curve
TF =: teriflunomide
TNF =: tumor necrosis factor
