# Supplementary material for: Cerebrospinal fluid cytokines after autologous haematopoietic stem cell transplantation and intrathecal rituximab treatment for multiple sclerosis
Source: Brain Commun. 2023 Jan 20;5(1):fcad011. doi: 10.1093/braincomms/fcad011 (PMC9901571; doi:10.1093/braincomms/fcad011)
Supplement: fcad011_Supplementary_Data [file fcad011_supplementary_data.pdf]

Supplementary Fig. 1

Protein concentrations of healthy controls, patients with progressive multiple sclerosis and patients with relapsing-remitting multiple sclerosis in the discovery and replication cohorts.

Using linear models adjusted for sex and age, 16 proteins were identified as the most important proteins differentiating between MS patients and healthy controls. Pairwise comparisons between groups were performed using the estimated marginal means from the linear regression model. Each datapoint represents a patient's protein NPX value. NS = not significant, *P < 0.05, **P < 0.01, ***P < 0.001

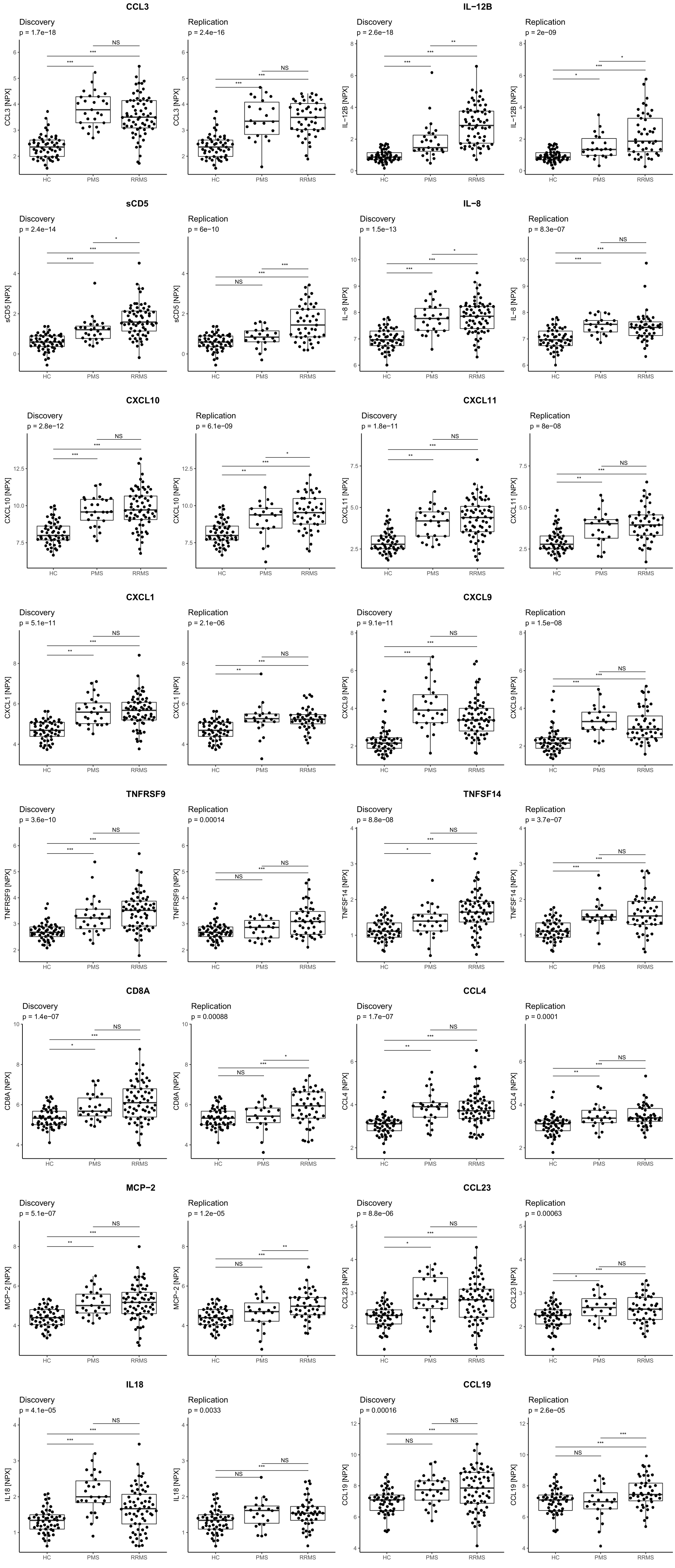

Supplementary Fig. 2

Protein concentrations depending on current treatment in relapsing-remitting multiple sclerosis patients from the combined discovery and replication cohorts.

Treatment effects on baseline were estimated using linear regression models adjusted for age and sex. Pairwise comparisons between treatments were estimated using the marginal means from the linear regression model. In general, patients with 1st line treatment had higher concentrations than untreated patients and patients treated with 2nd line treatment. Each datapoint represents a patient's protein NPX value. NS = not significant, *P < 0.05, **P < 0.01, ***P < 0.001

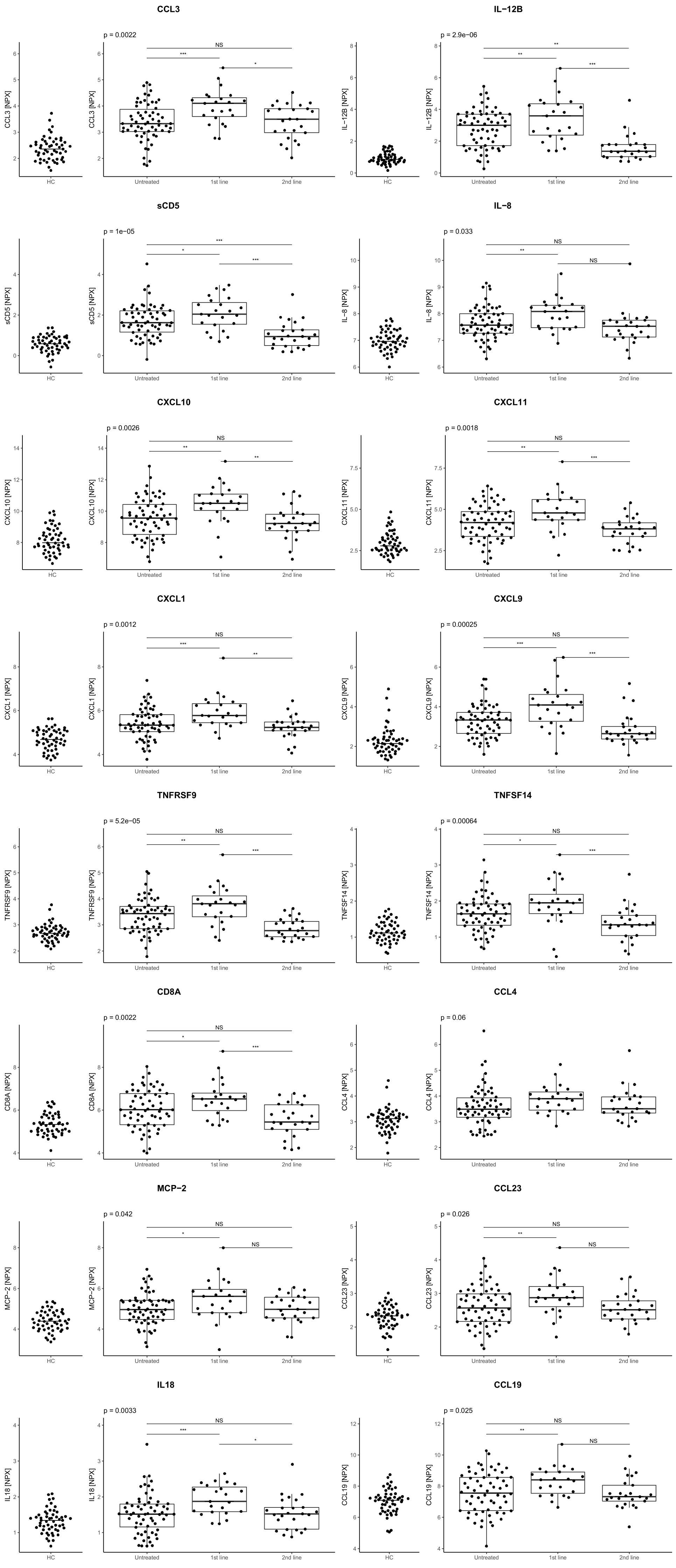

Supplementary Fig. 3

Protein concentrations after treatment intervention with autologous haematopoietic stem cell transplantation and an analysis of predictors for subsequent disease activity.

Linear mixed effects models adjusted for sex and age were used to estimate difference over time for the 16 proteins. Pairwise comparisons between time-points were estimated using the marginal means from the linear mixed linear models. The association with evidence of disease activity (EDA) was estimated within each time-point using the marginal means from the model. Each datapoint represents a patient's protein NPX value at the given time point. NS = not significant, *P < 0.05, **P < 0.01, ***P < 0.001

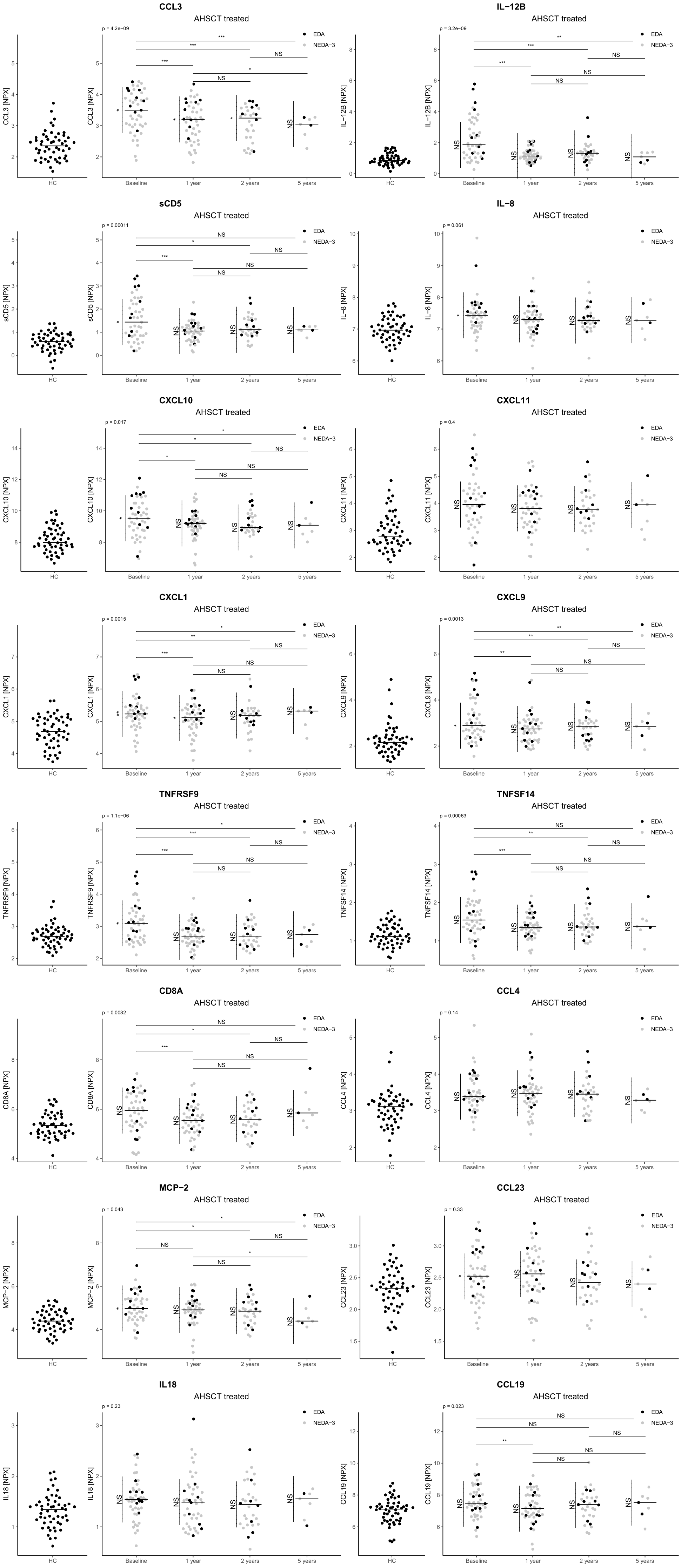

Supplementary Fig. 4

Protein concentrations after treatment intervention with intrathecal rituximab for progressive multiple sclerosis and an analysis of predictors for subsequent confirmed disability progression.

Linear mixed effects models adjusted for sex and age were used to estimate the difference over time for the 16 proteins. The association with disability progression was estimated within each time-point using the marginal means from the model. Each datapoint represents a patient's protein NPX value at the given time point. NS = not significant, *P < 0.05, **P < 0.01

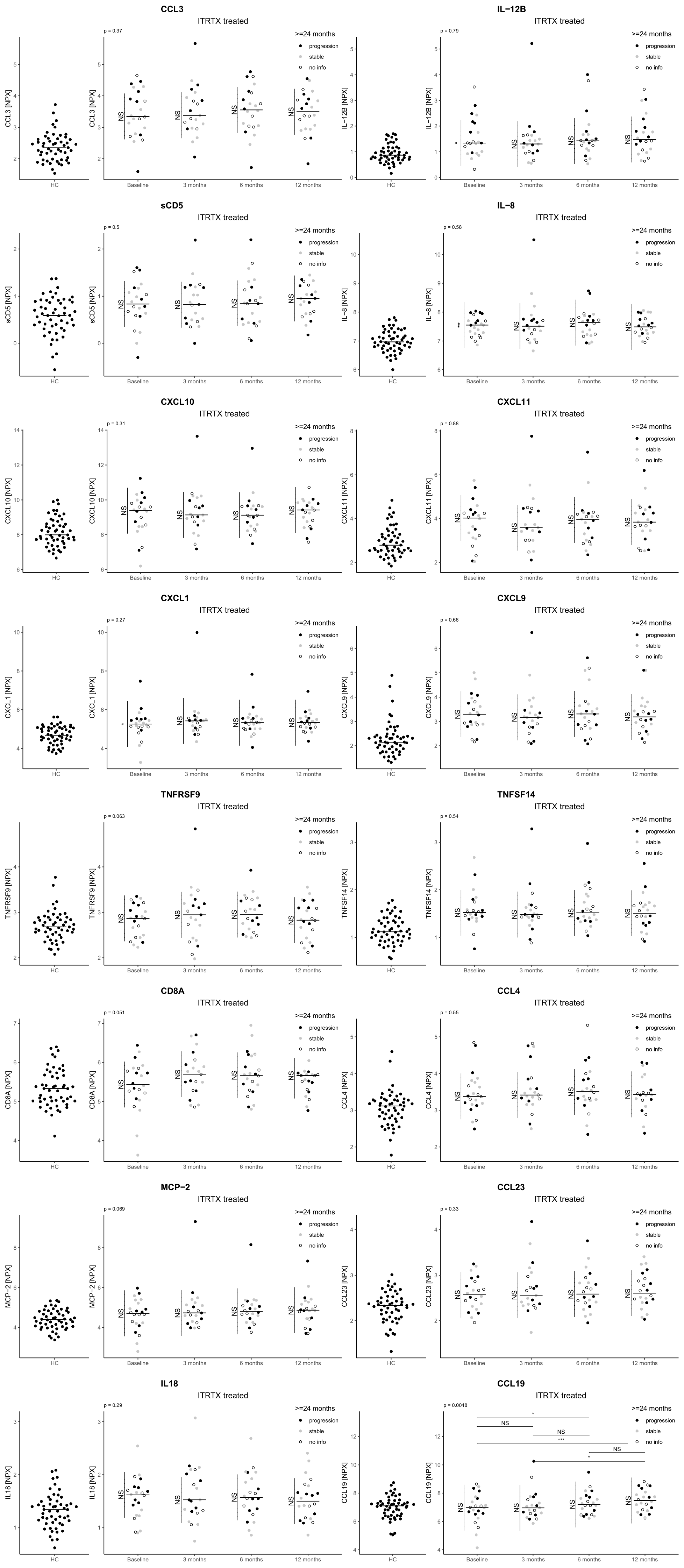

Supplementary Fig. 5

Receiver operating characteristic curves for prediction of subsequent disease activity in relapsing-remitting multiple sclerosis patients treated with autologous haematopoietic stem cell transplantation.

The discriminative ability of a protein or a combination of proteins was assessed using logistic regression and evaluated using receiver operating characteristic curve (ROC) area under the curve (AUC). The AUC is reported together with a 95% bootstrap confidence interval.

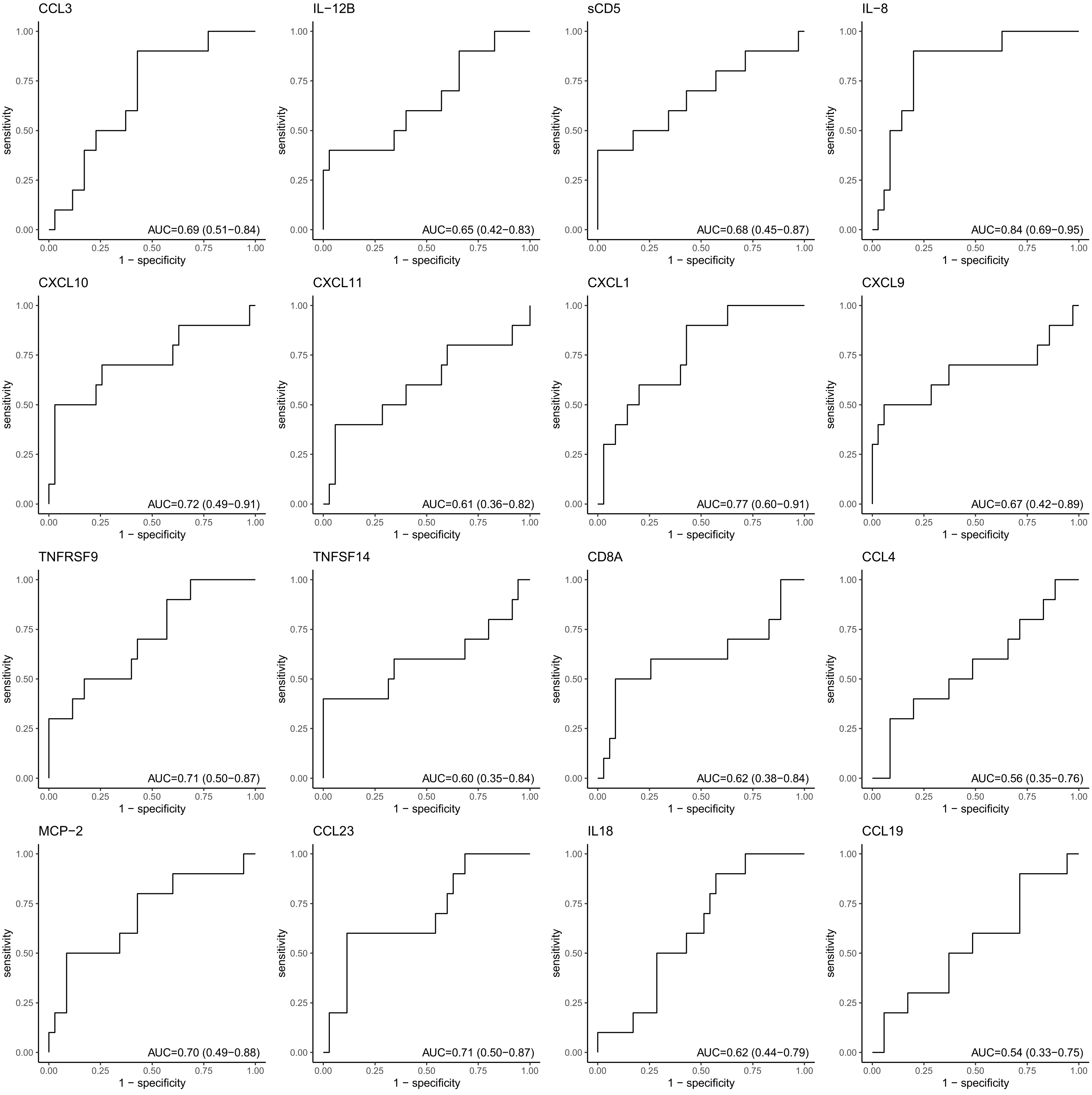

Supplementary Fig. 6

Receiver operating characteristic curves for prediction of subsequent confirmed disability worsening in patients treated with intrathecal rituximab for progressive multiple sclerosis.

The discriminative ability of a protein or a combination of proteins was assessed using logistic regression and evaluated using receiver operating characteristic curve (ROC) area under the curve (AUC). The AUC is reported together with a 95% bootstrap confidence interval.

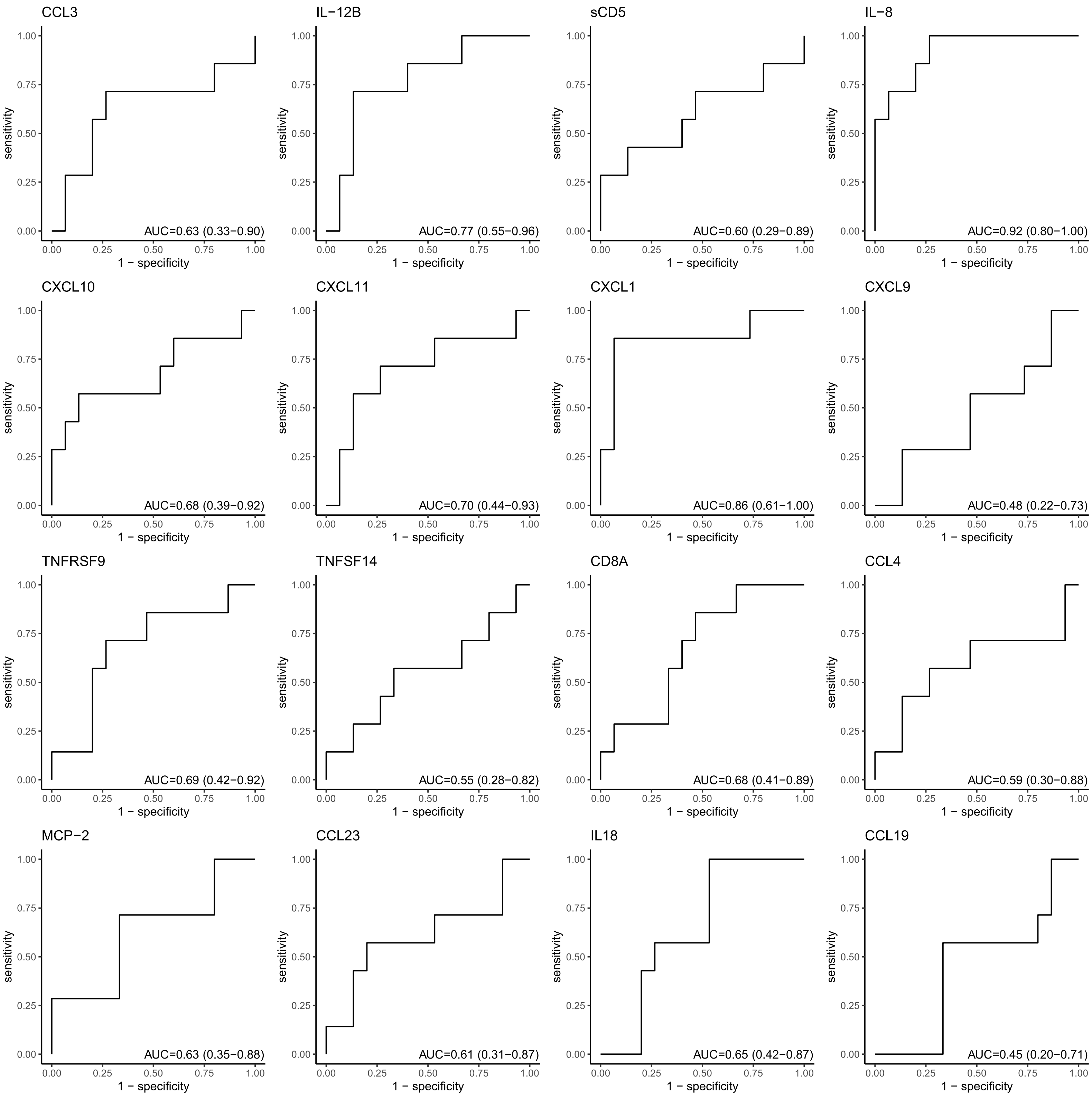

Supplementary Fig. 7

Receiver operating characteristic curves for prediction of subsequent disease activity in patients treated with intrathecal rituximab for progressive multiple sclerosis.

The discriminative ability of a protein or a combination of proteins was assessed using logistic regression and evaluated using receiver operating characteristic curve (ROC) area under the curve (AUC). The AUC is reported together with a 95% bootstrap confidence interval.

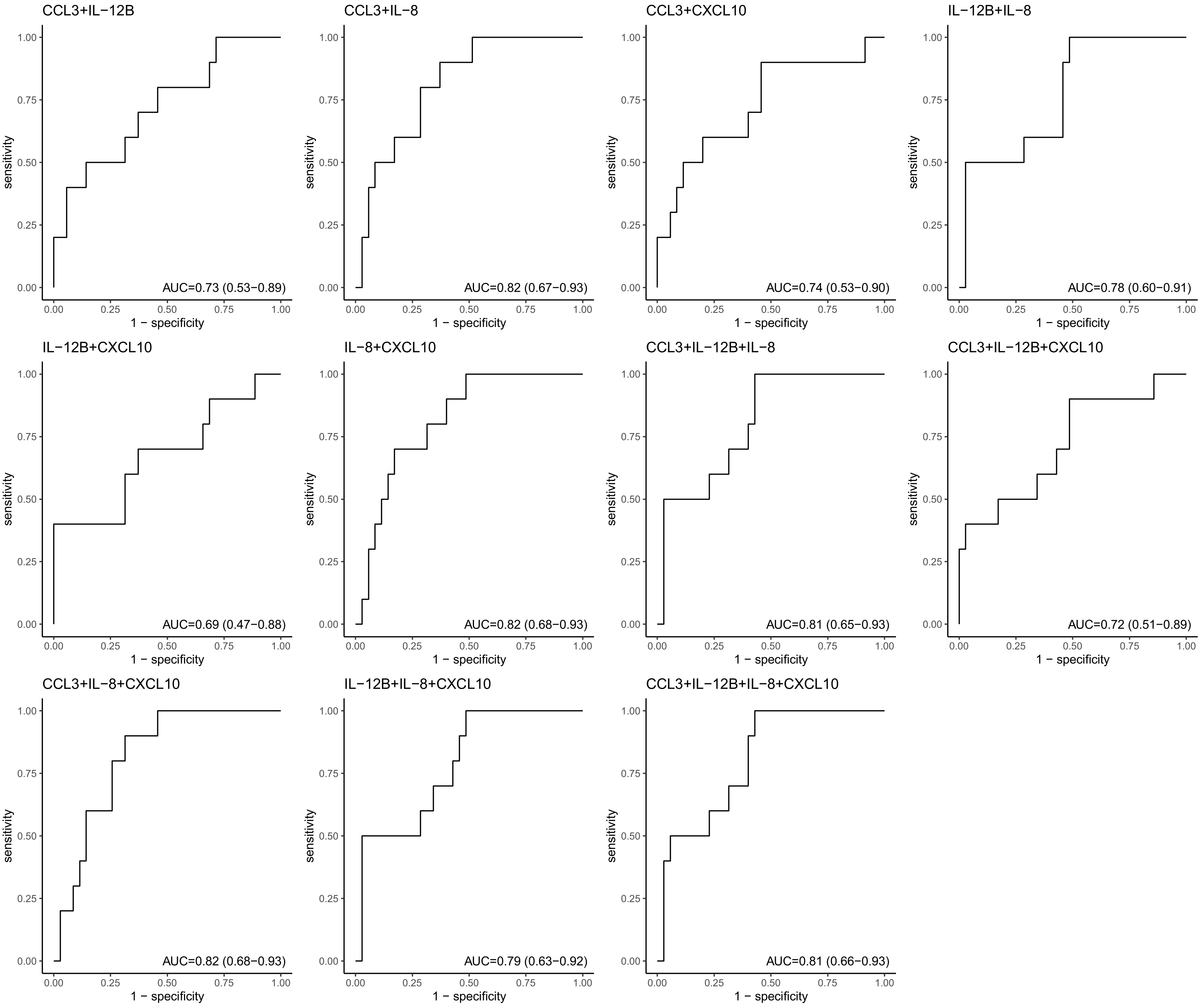

Supplementary Table 1: Comparison of 92 proteins between HC, PMS and RRMS in the discovery and replication cohorts.

The table display the results of the linear models adjusted for sex and age. Pairwise comparisons between groups were performed using the estimated marginal means from the linear regression model.

Discovery cohort

	Protein	ANOVA (P-value)	PMS vs HC (log2 fold-change)	PMS vs HC (P-value)	RRMS vs HC (log2 fold-change)	RRMS vs HC (P-value)	RRMS vs PMS (log2 fold-change)	RRMS vs PMS (P-value)	Age (log2 fold change)	Age (p-value)	Male vs Female (log2 fold change)	Male vs Female (p-value)
1	CCL3	1,74E-18	1,18373354	2,32E-09	1,20635832	2,82E-18	0,02262478	0,90168182	0,01106337	0,0171799	0,12317731	0,28022985
2	IL-12B	2,63E-18	1,14838426	8,65E-05	1,92028359	2,82E-19	0,77189932	0,00664557	-0,0102527	0,14699691	-0,4041246	0,021751
3	sCD5	2,40E-14	0,70494301	0,00029431	1,08822001	3,12E-15	0,38327699	0,04255669	-0,0046163	0,32764129	-0,1185402	0,31025666
4	IL-8	1,54E-13	0,52918717	0,00063322	0,83637315	1,96E-14	0,30718598	0,04152862	0,00972107	0,01049203	0,02186662	0,81410162
5	TNFB	2,29E-13	0,70932754	0,0007	1,12114679	2,93E-14	0,41181925	0,04320248	-0,0001368	0,97849124	-0,1839356	0,14488053
6	IFN-gamma	1,04E-12	1,31058009	0,00065772	1,99213544	1,44E-13	0,68155534	0,06831287	-0,0053398	0,5671893	-0,1342122	0,56150119
7	CXCL10	2,78E-12	1,16502206	0,00033303	1,63463015	4,63E-13	0,46960808	0,1350106	0,01291768	0,10163086	0,04884459	0,80175655
8	CXCL11	1,80E-11	0,88729755	0,00176386	1,38205354	2,46E-12	0,49475599	0,07364292	0,00892264	0,19736693	-0,1820585	0,2878203
9	CXCL1	5,07E-11	0,61641582	0,00145989	0,91770786	7,56E-12	0,30129203	0,10991466	0,01405397	0,00329304	-0,0857481	0,46259656
10	CXCL9	9,05E-11	1,18374103	1,16E-05	1,18569328	7,54E-11	0,00195224	0,99394216	0,02609755	8,39E-05	-0,1051722	0,51084677
11	TNFRSF9	3,63E-10	0,69535707	8,42E-05	0,76730257	1,54E-10	0,0719455	0,67158673	-0,0044581	0,29594446	0,01174547	0,91131037
12	TNF	5,78E-10	0,38177864	0,00044247	0,47534144	1,40E-10	0,0935628	0,3728172	0,00416074	0,11536831	-0,0071243	0,912944
13	CD6	5,72E-09	0,3054621	0,00582699	0,46391338	8,79E-10	0,15845128	0,14312713	0,00079185	0,76977589	-0,0397457	0,55332129
14	OSM	2,64E-08	0,27328324	0,00213308	0,35040591	5,78E-09	0,07712267	0,37221763	0,00612796	0,00527048	0,01291514	0,80977212
15	TNFSF14	8,81E-08	0,26273642	0,04754123	0,51379118	1,27E-08	0,25105476	0,05484288	-0,0012119	0,71010598	-0,0867014	0,28386663
16	CD8A	1,36E-07	0,50173705	0,02456882	0,84937152	2,03E-08	0,34763447	0,11269307	0,00138365	0,80050911	0,2409177	0,0773012
17	CCL4	1,73E-07	0,63078533	0,00137918	0,71762622	5,52E-08	0,08684089	0,64941034	0,00806779	0,09392316	-0,0156395	0,89518108
18	MCP-2	5,09E-07	0,62843693	0,00386264	0,77008551	1,27E-07	0,14164858	0,50311811	0,00493217	0,35320941	0,01837675	0,88876978
19	CCL23	8,79E-06	0,31284362	0,03537923	0,47741653	1,58E-06	0,16457291	0,25916877	0,01319515	0,00041109	0,15179765	0,09495562
20	CXCL6	1,07E-05	0,50882915	0,02993613	0,74380713	2,02E-06	0,23497798	0,30630943	0,02258573	0,00013167	0,25722802	0,0727182
21	IL-10RB	2,75E-05	0,35197865	0,00108147	0,29552051	2,77E-05	-0,0564581	0,58816712	0,00559532	0,0338071	0,05512413	0,39537678
22	CD40	2,83E-05	0,31588051	0,0096557	0,3599038	8,46E-06	0,04402329	0,7114184	0,01092089	0,00035078	0,02320873	0,75367973
23	IL18	4,11E-05	0,47895489	0,0007677	0,37243914	6,04E-05	-0,1065157	0,43938777	0,01267743	0,00033464	0,21418311	0,01326482
24	MCP-4	0,00014305	0,48644646	0,00543551	0,45338046	7,79E-05	-0,033066	0,84599606	0,01648034	0,00016955	0,36039919	0,0008431
25	CCL19	0,00015578	0,28759173	0,34521722	0,84126385	3,37E-05	0,55367211	0,06666105	0,01891605	0,01298517	0,53735296	0,00452117
26	IL10	0,00015847	0,09195614	0,51218533	0,38294232	4,15E-05	0,29098619	0,03682422	0,00390608	0,26144976	-0,0498786	0,56198894
27	OPG	0,00016239	0,26817834	0,06983072	0,4075823	3,25E-05	0,13940396	0,33734919	0,01932753	4,04E-07	0,31321771	0,00066909
28	Flt3L	0,00029666	0,25083248	0,03525684	0,31107934	7,58E-05	0,06024686	0,60552659	0,01531375	5,70E-07	0,0885365	0,2230815
29	Beta-NGF	0,00045382	0,04039521	0,31096172	0,10312231	9,64E-05	0,06272709	0,11169891	0,00223414	0,0246212	0,05123689	0,03721509
30	CCL28	0,00045925	0,25087459	0,00018329	0,10390134	0,0150199	-0,1469732	0,02395988	0,00348825	0,03256938	0,08671498	0,03195532
31	HGF	0,00046016	0,29766463	0,05175494	0,39044197	0,00010996	0,09277734	0,53634466	0,01821618	3,24E-06	0,13022992	0,16377872
32	CD244	0,00051273	0,14864236	0,15045306	0,26582437	0,00010234	0,117182	0,2498719	0,00697231	0,00695318	0,22856357	0,00040253
33	CDCP1	0,00069249	0,23493363	0,05982662	0,30970217	0,00016686	0,07476855	0,54143261	0,01103686	0,00043957	0,1674128	0,02908084
34	LIF	0,00083919	0,13726479	0,13733927	0,22904468	0,00017451	0,09177988	0,31287787	0,00262376	0,25054177	0,1467823	0,01015245
35	IL-18R1	0,00209691	0,30331153	0,04848636	0,34391756	0,00064397	0,04060603	0,78746042	0,00650513	0,0868367	-0,003378	0,97121261
36	CCL11	0,00243341	0,222608	0,02059362	0,20309351	0,00120659	-0,0195145	0,83544176	0,00575936	0,01562074	0,09701643	0,09824617
37	CCL20	0,00301703	0,1298158	0,23111158	0,24274471	0,00067175	0,11292891	0,29069337	0,00966054	0,00041739	0,17938573	0,00756083
38	MMP-10	0,00308811	0,21560206	0,09273429	0,28229035	0,00079841	0,06668828	0,59643241	0,00701402	0,02771957	0,22851765	0,00400886
39	CSF-1	0,00381932	0,08669892	0,45759239	0,25399471	0,00095394	0,16729579	0,1473471	0,01233046	3,46E-05	0,09497441	0,18544422
40	IL-1 alpha	0,00427549	0,42440902	0,00841635	0,29199091	0,00511837	-0,1324181	0,39935988	4,08E-05	0,99174154	0,09309702	0,34065161
41	STAMBP	0,00431561	0,01938264	0,76467487	0,1358067	0,00144265	0,11642405	0,06994244	0,00595696	0,00028391	0,01732037	0,66271853
42	IL6	0,00482761	0,34241941	0,06899438	0,39142489	0,00149215	0,04900548	0,79073835	0,0035269	0,44707211	0,10178031	0,37589364
43	uPA	0,0050531	0,31648288	0,02782245	0,28285277	0,00254706	-0,0336301	0,81110533	0,0030446	0,38915346	0,16251985	0,0647521
44	TGF-alpha	0,00526755	-0,1013991	0,4141404	0,21774941	0,00734186	0,31914847	0,00994734	0,01017654	0,00114581	0,03343203	0,66032073
45	4E-BP1	0,00620261	0,1076284	0,39852687	0,26578415	0,0015192	0,15815575	0,20924188	0,01019549	0,00149106	0,08822272	0,25954972
46	CXCL5	0,00665346	0,05861109	0,69291027	0,30069188	0,00205948	0,24208079	0,09978802	0,01460513	0,00010882	-0,0011732	0,98971166
47	TRAIL	0,00877938	0,14494912	0,16436054	0,20853259	0,00226219	0,06358347	0,53521799	0,0067007	0,01002957	0,18262739	0,00470982
48	FGF-5	0,02083423	-0,2894439	0,0493799	0,1135446	0,23102406	0,40298853	0,00586632	0,00877397	0,0164709	0,09643976	0,28322433
49	CCL25	0,02444057	0,16975279	0,08183804	0,16317965	0,01014792	-0,0065731	0,94524788	0,00960903	9,92E-05	-0,0022159	0,97028895
50	TWEAK	0,03167102	0,09556319	0,49551668	0,23952366	0,00899295	0,14396047	0,29850153	0,01354834	0,00014146	0,12374403	0,15123916
51	LAP TGF-beta-1	0,03557512	-0,3569947	0,03162053	0,06597111	0,53590669	0,42296578	0,01011266	0,03381279	7,64E-14	0,12182207	0,22902575
52	PD-L1	0,03559885	0,03109106	0,82192063	0,22487254	0,01276199	0,19378149	0,1564627	0,0177745	6,70E-07	0,15314012	0,07222954
53	NRTN	0,03927342	-0,1370089	0,01516862	-0,0046688	0,89707585	0,1323401	0,01737183	0,00185684	0,18048899	0,0045996	0,89311682
54	VEGFA	0,0449921	-0,1242814	0,39621821	0,18248062	0,05528526	0,30676198	0,0349781	0,0103279	0,00493378	0,0762595	0,39589799

55 FGF-19	0,04822416	-0,3897569	0,02036807	-0,0027788	0,97939487	0,38697808	0,01958371	0,01270598	0,00241794	0,21208697	0,03915641
56 SIRT2	0,05120982	0,10842043	0,34753367	0,18308571	0,01503268	0,07466527	0,51162088	0,01183877	5,58E-05	-0,0218765	0,7569044
57 LIF-R	0,05963533	-0,176513	0,1180018	0,08412133	0,24823772	0,26063431	0,01994675	0,01224623	2,06E-05	0,09325021	0,1775611
58 SCF	0,06905877	0,1692925	0,1749129	0,18045382	0,02613227	0,01116133	0,92752236	0,01067002	0,00068883	0,14705184	0,05544106
59 MCP-1	0,11524498	0,32016879	0,04462539	0,12219267	0,23362444	-0,1979761	0,20612827	0,01166773	0,00336855	0,29338442	0,00292975
60 ARTN	0,11682727	-0,0732473	0,28975658	0,05892317	0,18822456	0,13217044	0,05392277	0,00241522	0,15915967	0,06391936	0,13270024
61 DNER	0,15914295	-0,0544301	0,2514306	0,03232573	0,29190582	0,08675579	0,06487691	0,00468085	0,00010192	0,04065765	0,1628586
62 CASP-8	0,16310353	-0,0558322	0,34594374	0,04733664	0,21698071	0,10316882	0,07867013	0,00375725	0,01119355	0,02515919	0,48823086
63 EN-RAGE	0,16855333	0,00841673	0,88817732	0,07056381	0,0698661	0,06214709	0,2935243	-0,0004974	0,73715491	0,0248716	0,49834988
64 IL13	0,18832223	-0,1776412	0,06868345	-0,0463322	0,46044794	0,13130901	0,17141896	0,00254693	0,28982001	0,04470153	0,45277742
65 IL-17A	0,20956391	0,06367718	0,47257808	0,10139451	0,07824595	0,03771733	0,66607324	0,00039896	0,8556256	-0,0418373	0,44157642
66 MCP-3	0,2206946	0,01014475	0,91580021	0,10369097	0,09625691	0,09354621	0,32385561	0,00266366	0,263202	-0,0500364	0,39569575
67 TRANCE	0,23718684	-0,1334766	0,10348671	-0,0064063	0,90333541	0,12707025	0,11600082	0,00139771	0,48931261	-0,0021309	0,96602688
68 MMP-1	0,26080636	0,2998402	0,12990898	-0,0082243	0,94855753	-0,3080645	0,11484685	-0,0041928	0,39097374	0,00228258	0,98493732
69 IL-20	0,28609638	-0,0780668	0,1371068	-0,0003047	0,99281086	0,0777621	0,1333489	0,00108317	0,4034434	0,01668165	0,60314955
70 IL5	0,30653385	-0,1627103	0,25692792	0,05436625	0,55726491	0,21707653	0,12594282	0,00401793	0,25811641	-0,0372848	0,67120251
71 IL7	0,34973119	-0,0143346	0,86795359	-0,0787006	0,15943179	-0,064366	0,44962689	0,00564607	0,00897228	0,03881845	0,46320475
72 IL-20RA	0,35078938	0,01790722	0,75707012	0,05376685	0,15225992	0,03585963	0,53026982	-5,84E-05	0,96750499	0,01025686	0,77261814
73 NT-3	0,38306466	-0,0888969	0,20338778	0,00332694	0,941134	0,09222382	0,18114616	0,00102118	0,55414611	-0,0282642	0,50866257
74 IL-24	0,39240895	0,14245338	0,31596171	0,11241009	0,22371048	-0,0300433	0,83016151	-0,0033104	0,34644762	0,06579343	0,45319251
75 IL-2RB	0,39451493	-0,1149926	0,17479338	-0,0198679	0,71603863	0,09512472	0,254701	0,00222675	0,28787778	0,01202892	0,81635727
76 ST1A1	0,4294534	0,01601931	0,84259165	0,06656035	0,20314265	0,05054104	0,52562941	0,00279668	0,1628249	0,06738893	0,17448196
77 CX3CL1	0,43945982	-0,0734835	0,56709999	0,07315993	0,37849769	0,14664342	0,24780584	0,00703034	0,02822905	0,04210364	0,59276879
78 IL2	0,46969793	-0,0794079	0,2649961	0,00459699	0,92031789	0,08400488	0,23210677	0,00198692	0,25991961	0,05080394	0,24491815
79 ADA	0,55435537	0,0082682	0,95934707	0,10871225	0,30081553	0,10044405	0,53047748	0,00716066	0,07619592	0,13535417	0,17498254
80 TSLP	0,57673037	0,00734084	0,94898691	-0,0713193	0,33711411	-0,0786602	0,48745524	0,00084984	0,76482623	-0,1391587	0,04949684
81 FGF-23	0,58701786	-0,012563	0,90329268	-0,0671353	0,31607774	-0,0545723	0,59281018	-0,0027421	0,28501845	-0,1317036	0,03924973
82 IL-10RA	0,64375045	-0,0276146	0,54414604	0,01362276	0,64338877	0,04123736	0,35892668	0,00075494	0,50302679	0,01675507	0,54837733
83 CST5	0,73394216	0,02074934	0,47293335	-0,0009779	0,95823498	-0,0217272	0,44620986	0,00144817	0,04436108	0,03702726	0,0380296
84 SLAMF1	0,75676548	0,03018867	0,75042293	0,04558255	0,45790915	0,01539389	0,86938359	0,00056863	0,80875223	0,04198285	0,47099826
85 FGF-21	0,76408406	-0,0739248	0,46695764	-0,011249	0,86393087	0,0626758	0,53174065	0,00704736	0,00568163	-0,0222004	0,72144894
86 IL4	0,81327861	-0,0498126	0,56985119	-0,0269609	0,63419009	0,02285165	0,79148116	-0,001329	0,54026868	0,00368947	0,94524378
87 IL-15RA	0,84286889	-0,0181274	0,76878106	0,01485461	0,70946115	0,03298203	0,58782373	0,00320451	0,03723126	-0,0237783	0,5296685
88 GDNF	0,86611731	-0,0445561	0,62564965	0,00229853	0,96893402	0,0468546	0,60304531	0,00270556	0,23243548	0,04365653	0,43595919
89 IL33	0,8749227	-0,0196568	0,74763622	0,01059675	0,78845653	0,03025357	0,61576186	-0,0003492	0,81736922	-0,0432762	0,24903085
90 IL-22 RA1	0,89372314	-0,0733956	0,63614983	-0,0172865	0,86311498	0,05610914	0,7138584	0,00167217	0,66326203	-0,0579535	0,54249918
91 IL-17C	0,92485471	-0,0288545	0,69592868	-0,0088475	0,85291472	0,02000697	0,78351783	0,00026316	0,88548771	0,00667419	0,882769
92 AXIN1	0,98809378	0,00809328	0,89344164	0,00462383	0,90577114	-0,0034695	0,95358075	0,00049052	0,74299523	0,00115904	0,97504213

Validation cohort

Protein	ANOVA (P-value)	PMS vs HC (log2 fold-change)	PMS vs HC (P-value)	RRMS vs HC (log2 fold-change)	RRMS vs HC (P-value)	RRMS vs PMS (log2 fold-change)	RRMS vs PMS (P-value)	Age (log2 fold change)	Age (p-value)	Male vs Female (log2 fold change)	Male vs Female (p-value)
1 CCL3	2,40E-16	0,94922736	2,21E-08	1,13829547	5,88E-16	0,18906811	0,26212395	0,01223324	0,01152011	0,11361959	0,30945737
2 IL-12B	1,97E-09	0,63674689	0,01117684	1,30505692	2,94E-10	0,66831003	0,01218412	-0,0042176	0,57246672	-0,2342184	0,18106696
3 sCD5	6,01E-10	0,24266954	0,16470987	0,94671215	9,69E-11	0,70404261	0,00021864	-0,0013121	0,80260545	-0,1484132	0,22755029
4 IL-8	8,34E-07	0,41762138	0,00075636	0,4868678	6,44E-07	0,06924642	0,59021718	0,00837563	0,0232391	-0,0261541	0,7590383
5 TNFB	1,52E-07	0,3351628	0,04680056	0,76645252	2,30E-08	0,43128972	0,01648434	0,00226369	0,6536843	-0,1198327	0,31018191
6 IFN-gamma	7,60E-10	1,13497554	0,00062726	1,71920628	2,25E-10	0,58423074	0,09120679	0,01068006	0,2751427	-0,2657636	0,24520834
7 CXCL10	6,05E-09	0,74016081	0,00808347	1,39847496	1,02E-09	0,65831415	0,02595503	0,01713369	0,04090488	-0,202023	0,29881911
8 CXCL11	8,00E-08	0,669984	0,00527857	1,09134015	1,84E-08	0,42135615	0,09508442	0,01197967	0,09469155	-0,2994152	0,07403115
9 CXCL1	2,13E-06	0,41513211	0,00520712	0,58518391	7,20E-07	0,1700518	0,27449106	0,01056227	0,01792277	-0,2116854	0,04161076
10 CXCL9	1,50E-08	0,77454898	0,000142	0,92148222	1,34E-08	0,14693323	0,48362614	0,02567065	3,28E-05	-0,0480987	0,72938384
11 TNFRSF9	0,00013833	0,17753844	0,15168853	0,41262336	2,62E-05	0,23508492	0,07474919	-0,0050891	0,17306995	0,09867674	0,25752856
12 TNF	8,33E-10	0,24127877	0,0021704	0,41316709	1,68E-10	0,17188832	0,03766053	0,00493489	0,03564114	-0,0287498	0,59699512
13 CD6	3,36E-05	0,04982043	0,55063757	0,29793164	8,03E-06	0,24811122	0,00590551	0,00343587	0,17397899	-0,0693209	0,2398444
14 OSM	4,61E-06	0,27396922	0,00042352	0,27046807	7,83E-06	-0,0035011	0,96524951	0,00456839	0,0471769	-0,0505753	0,34371265

15 TNFSF14	3,66E-07	0,3859	0,00067912	0,46342696	2,55E-07	0,07752695	0,51034844	0,00319862	0,339355	-0,1207985	0,12363408
16 CD8A	0,00087943	0,07521289	0,68061475	0,52946517	0,00023728	0,45425228	0,02071775	-3,78E-05	0,99453106	0,1189418	0,35649828
17 CCL4	0,00010182	0,35892661	0,00968509	0,44158966	4,75E-05	0,08266305	0,56966783	0,00398576	0,33500482	-0,0294398	0,7600728
18 MCP-2	1,23E-05	0,09652815	0,57178479	0,63973577	3,05E-06	0,54320761	0,00328101	0,00985847	0,05743718	-0,1090185	0,36551008
19 CCL23	0,00062906	0,19495722	0,0413187	0,2783328	0,0001958	0,08337557	0,40800565	0,01012641	0,00055562	0,09240199	0,16800344
20 CXCL6	0,01083029	0,00652837	0,96941904	0,3824307	0,00396724	0,37590234	0,03957505	0,01863664	0,00041755	0,13028853	0,27898426
21 IL-10RB	0,69978798	-0,0074223	0,92615467	0,04741393	0,43985362	0,05483622	0,51969199	0,00166663	0,49083572	0,04091135	0,46912327
22 CD40	0,93300226	-0,0048172	0,96055074	0,02511528	0,7362726	0,02993248	0,77244682	0,00506076	0,08707278	-0,0045777	0,94683781
23 IL18	0,00334961	0,15022755	0,12527116	0,25450339	0,00087449	0,10427584	0,31522555	0,00756706	0,01120779	0,05192945	0,45044091
24 MCP-4	0,07613225	0,088556	0,51713581	0,23942023	0,02355601	0,15086423	0,29973434	0,01244655	0,00305396	0,17607519	0,069428
25 CCL19	2,55E-05	-0,2569859	0,27294819	0,74097989	6,42E-05	0,99796576	0,00010218	0,01987708	0,00559885	0,55211929	0,00106745
26 IL10	0,00118829	0,03424495	0,78243624	0,34863145	0,00035514	0,31438649	0,01841061	0,00346326	0,35552995	-0,0840877	0,33698661
27 OPG	0,70562531	-0,0985059	0,42879902	-0,0462972	0,62681752	0,05220869	0,69280459	0,0138414	0,00033521	0,26305169	0,00323019
28 Flt3L	0,01719374	-0,061725	0,4749718	0,16282478	0,01498164	0,22454974	0,01563396	0,01135048	2,76E-05	0,06250885	0,30530307
29 Beta-NGF	0,3260466	-0,0376666	0,15750282	-0,0180608	0,37468492	0,01960577	0,48725733	0,0002001	0,80265855	0,03062091	0,10356442
30 CCL28	0,05750774	0,04517621	0,47927353	0,11790246	0,01709261	0,07272624	0,28449748	0,00460471	0,01811668	0,11398293	0,01240267
31 HGF	0,08470548	-0,0349611	0,75614789	0,17454553	0,04464754	0,20950665	0,08184737	0,01414564	5,85E-05	0,13907351	0,08164488
32 CD244	0,11618059	-0,1138415	0,18829691	0,07601903	0,25068976	0,18986051	0,04001332	0,00653787	0,01314843	0,2383922	0,0001446
33 CDCP1	0,51728017	-0,0132782	0,89647835	0,08208569	0,29451987	0,09536386	0,37998033	0,00734602	0,01843826	0,17583288	0,01580873
34 LIF	0,11823176	0,03921688	0,54238121	0,10247503	0,03921303	0,06325815	0,35559635	0,0025949	0,18293406	0,09348826	0,04103762
35 IL-18R1	0,03516329	0,05432078	0,68255743	0,26345265	0,01061237	0,20913187	0,14015036	0,00446168	0,26673626	0,03476911	0,71035777
36 CCL11	0,49805256	0,03844083	0,52014543	0,05202143	0,25647713	0,01358059	0,83055903	0,00318973	0,07872388	0,08303409	0,05046263
37 CCL20	0,59415162	0,03774294	0,70767644	0,07839544	0,30988224	0,0406525	0,70388961	0,00829451	0,00717192	0,17836534	0,01310356
38 MMP-10	0,00771504	-0,0388972	0,73874975	0,26048512	0,00415503	0,29938228	0,01702035	0,00098321	0,77994536	0,24210789	0,00382846
39 CSF-1	0,22643157	-0,1482125	0,09077446	-0,0137638	0,83644227	0,13444869	0,14810503	0,00783612	0,00342885	0,0836074	0,17494186
40 IL-1 alpha	0,33980739	-0,0169307	0,86221853	0,10038535	0,18055183	0,11731604	0,25906122	0,00310725	0,29231991	-0,0112106	0,87050183
41 STAMBP	0,49219804	0,04848916	0,36467074	0,04118378	0,3147249	-0,0073054	0,89755605	0,00366112	0,0246225	0,01855739	0,62217044
42 IL6	0,02278289	0,18991489	0,28478877	0,37667705	0,00627365	0,18676216	0,32206704	0,00602069	0,2612519	0,00373952	0,97610494
43 uPA	0,37386032	0,03794644	0,66972085	0,09569571	0,16160273	0,05774927	0,54146096	-0,0018491	0,49123589	0,07018938	0,26416104
44 TGF-alpha	0,05539501	-0,1947631	0,04495211	-0,1393124	0,0607056	0,05545075	0,58808005	0,00532659	0,0687436	-0,0042724	0,94980862
45 4E-BP1	0,15901159	0,02930063	0,74630315	0,13249844	0,05788764	0,1031978	0,2846894	0,00385942	0,15960679	0,02563748	0,68803993
46 CXCL5	0,01326068	0,0900161	0,37244079	0,23060554	0,00334574	0,14058944	0,19077961	0,01264619	5,96E-05	-0,0520732	0,46395164
47 TRAIL	0,30496725	-0,0815323	0,33967508	0,05697671	0,38325196	0,13850905	0,12806964	0,00329438	0,20177896	0,08444964	0,16158922
48 FGF-5	0,00096185	-0,3981737	0,00205507	-0,2934428	0,00296205	0,10473092	0,43658788	0,00221066	0,56284729	0,0645222	0,46990311
49 CCL25	0,23552652	0,07367629	0,34039619	0,09658558	0,10384501	0,02290929	0,77987476	0,01083157	8,42E-06	0,03624125	0,50544014
50 TWEAK	0,10857875	-0,2214831	0,04493922	-0,1021492	0,22441741	0,11933383	0,30613709	0,0076534	0,02201629	0,14074833	0,07019593
51 LAP TGF-beta-1	7,92E-07	0,0792777	0,52809674	-0,4950154	1,03E-06	-0,5742931	3,41E-05	0,03251691	4,61E-14	0,06359297	0,47290686
52 PD-L1	0,26349033	-0,1695506	0,10490386	-0,05125	0,51997158	0,11830056	0,28544204	0,00961916	0,00264741	0,11585584	0,11586573
53 NRTN	0,29005349	-0,0702214	0,14213365	0,0035253	0,92295057	0,07374672	0,14681511	0,00182882	0,2046549	0,00729616	0,82792783
54 VEGFA	0,00523866	-0,3423837	0,00417843	-0,2123203	0,01956981	0,13006339	0,29828742	0,00375767	0,29000014	0,06996572	0,39859997
55 FGF-19	0,0003111	-0,528461	0,00013442	-0,2490522	0,01652681	0,27940885	0,05169606	0,00609951	0,13342136	0,14788775	0,11946164
56 SIRT2	0,61471345	-0,0905461	0,37895659	-0,0539468	0,49320631	0,03659929	0,73751178	0,00754716	0,01622857	0,04469683	0,53746931
57 LIF-R	0,00081634	-0,2652123	0,00370554	-0,2255145	0,00132585	0,03969776	0,67720898	0,00691735	0,0117355	0,07068332	0,26497454
58 SCF	0,47733194	-0,1045185	0,30137058	0,02327651	0,76317945	0,12779505	0,23472247	0,00701816	0,02266369	0,17170851	0,01710866
59 MCP-1	0,11656577	0,07193347	0,54055244	0,18774777	0,03856584	0,11581431	0,35432114	0,00954213	0,00801909	0,12862849	0,12220738
60 ARTN	0,29988264	0,04200647	0,47782938	-0,0502337	0,26825973	-0,0922402	0,14387323	0,00231167	0,19664455	-0,011909	0,77508629
61 DNER	0,02967205	-0,1172818	0,01496984	-0,0640307	0,0807676	0,05325113	0,29341547	0,0026662	0,06529946	0,05572702	0,09861922
62 CASP-8	0,01384346	0,01327021	0,77204982	-0,0960324	0,00702205	-0,1093026	0,02630069	0,00130082	0,34751079	0,02371421	0,46314965
63 EN-RAGE	0,03209253	0,29165548	0,0173801	-0,0326237	0,72498994	-0,3242791	0,01292309	-0,0021098	0,56401409	-0,1152837	0,17860751
64 IL13	0,0166621	-0,0255743	0,73011254	-0,1621489	0,00497051	-0,1365746	0,08490398	0,00115029	0,60722366	-0,0365086	0,48514095
65 IL-17A	0,13753382	-0,0500989	0,38652729	0,06483975	0,14439797	0,11493864	0,0630954	0,00185313	0,28887508	-0,0336727	0,40893538
66 MCP-3	0,37151276	0,14479819	0,18265573	-0,0022153	0,97866948	-0,1470135	0,20277476	0,00548024	0,09536605	0,03878825	0,61138673
67 TRANCE	0,84229013	-0,0145031	0,8483706	0,0273288	0,63802947	0,04183185	0,6039623	0,00092315	0,68680328	0,05300439	0,32254472
68 MMP-1	0,24451908	-0,0599279	0,6064135	-0,1499501	0,09399699	-0,0900222	0,46668598	-0,0101772	0,00437335	0,06399288	0,43547621
69 IL-20	0,68487983	0,00749704	0,86179495	-0,0250938	0,44708012	-0,0325908	0,47680679	-9,66E-05	0,94076388	0,00809622	0,78971685
70 IL5	0,05947308	-0,0916827	0,14970667	-0,1095718	0,02543833	-0,0178891	0,79048861	0,00217707	0,25614216	-0,0731504	0,10342869
71 IL7	0,2060616	-0,0551898	0,46295778	-0,1019176	0,07828328	-0,0467278	0,558503	0,00052975	0,81520019	-0,0236109	0,65575339
72 IL-20RA	0,61388015	-0,0118542	0,81593586	-0,0384234	0,32528229	-0,0265693	0,62358497	-0,0017857	0,2465551	0,06660176	0,06550542
73 NT-3	0,11282742	-0,0080421	0,89772289	-0,0978506	0,04288119	-0,0898085	0,17845319	0,00129147	0,49436637	-0,0277947	0,52889791

74	IL-24	0,36153948	0,17067306	0,1684303	0,00630684	0,94677359	-0,1643662	0,21162547	-0,0005271	0,88745279	-0,0284024	0,74411181
75	IL-2RB	0,25732162	-0,03529	0,61058988	-0,087683	0,10017062	-0,052393	0,47703392	0,00388576	0,06497742	-0,0300791	0,53817921
76	ST1A1	0,79423772	0,04605027	0,50251537	0,01531649	0,77061412	-0,0307338	0,67347342	0,00498317	0,01743878	0,06907162	0,15506522
77	CX3CL1	0,06185527	-0,2553487	0,02287243	-0,1083667	0,20357694	0,14698207	0,21405418	0,00381321	0,25605384	0,06268902	0,42346105
78	IL2	0,96959856	-0,0109326	0,86206763	-0,0106309	0,82533324	0,00030174	0,99639926	0,00205187	0,28105057	0,005383	0,90340426
79	ADA	0,00149394	-0,475171	0,00048193	-0,1984303	0,05239138	0,27674072	0,05131376	0,00131117	0,74306841	0,09536775	0,30842338
80	TSLP	0,8991295	-0,0007907	0,99409429	-0,036108	0,65895722	-0,0353173	0,75576347	0,00197617	0,54018644	-0,1185505	0,11738085
81	FGF-23	0,07739373	-0,0945786	0,31062708	-0,1605989	0,02576791	-0,0660203	0,50484509	-0,0004734	0,86615636	-0,1380814	0,03709598
82	IL-10RA	0,65417711	-0,0341653	0,36642016	-0,0030635	0,91559857	0,03110182	0,4388632	0,00104026	0,36212658	0,00733861	0,78280881
83	CST5	0,16757166	-0,0314886	0,18289227	0,01605468	0,37399979	0,04754323	0,05941844	0,00058224	0,41329495	0,04518333	0,00737992
84	SLAMF1	0,91108371	0,01231546	0,88514496	-0,0224675	0,73071464	-0,0347829	0,70111074	-0,0001337	0,95856436	-0,0542979	0,36710865
85	FGF-21	0,48779749	-0,0825606	0,38264263	-0,0754754	0,29747757	0,00708522	0,94369779	0,00903639	0,00190017	0,01890236	0,7764282
86	IL4	0,37920539	-0,0305454	0,68117309	0,06543443	0,25142937	0,09597983	0,2257138	-0,0021302	0,34312497	-0,0183285	0,72651084
87	IL-15RA	0,82134485	-0,0200583	0,72310397	-0,0259478	0,54954369	-0,0058895	0,92199113	0,00262844	0,1257228	0,00403128	0,91951619
88	GDNF	0,15748446	-0,0160254	0,85518141	-0,1271753	0,06042798	-0,1111499	0,2349595	0,00139409	0,59897434	0,02103707	0,73399181
89	IL33	0,131035	0,0883469	0,12096296	-0,0336358	0,43874815	-0,1219827	0,04466772	0,00093531	0,58467334	-0,0381252	0,34086651
90	IL-22 RA1	0,09838061	-0,296493	0,03166011	-0,0645601	0,5373155	0,23193292	0,1120583	0,00295609	0,47378096	-0,1601347	0,09831204
91	IL-17C	0,37148036	-0,0109977	0,87181525	-0,0722804	0,16769808	-0,0612827	0,39819698	0,00037842	0,85402308	0,01317123	0,78400424
92	AXIN1	0,12953896	0,04931944	0,27762086	-0,0456753	0,1895546	-0,0949948	0,0504067	0,00037295	0,78506425	-0,0014433	0,96395699

Supplementary Table 2: Comparison of previous treatment in RRMS patients from combined discovery and replication cohorts.

Treatment effects on baseline were estimated using linear regression models adjusted for age and sex for the 16 proteins. Pairwise comparisons between treatments were estimated using the marginal means from the linear regression model.

	Protein	ANOVA (p-value)	ANOVA (q-value)	1st line vs 2nd line (log2 fold change)	1st line vs 2nd line (p-value)	1st line vs untreated (log2 fold change)	1st line vs untreated (p-value)	Age (log2 fold change)	Age (p-value)	Male vs Female (log2 fold change)	Male vs Female (p-value)
1	IL-12B	2,94E-06	4,71E-05	1,847681944	4,95E-07	0,900412718	0,003001038	-0,035009637	0,009275819	-0,252512181	0,290604283
2	sCD5	1,02E-05	8,16E-05	1,168703129	1,94E-06	0,432651117	0,032192137	-0,019170826	0,033194631	-0,112470082	0,483371381
3	TNFRSF9	5,17E-05	0,000275781	0,840528758	1,57E-05	0,52531657	0,001358299	-0,011991974	0,094757266	0,141373797	0,272041904
4	CXCL9	0,000251621	0,001006482	1,036021057	0,000221158	0,825231628	0,000590625	0,005450096	0,600546638	-0,039113659	0,834558532
5	TNFSF14	0,000639381	0,002046018	0,610899893	0,00014697	0,331642651	0,01444777	-0,011456869	0,056571521	-0,199158733	0,065453769
6	CXCL1	0,001240186	0,003307164	0,524668135	0,008572872	0,618029829	0,000385339	0,012763486	0,092076912	0,079549884	0,557376993
7	CXCL11	0,001847283	0,003972327	1,102330911	0,00093721	0,809751823	0,004434142	-0,008481963	0,495719595	-0,184579054	0,410467682
8	CD8A	0,002217736	0,003972327	0,937220506	0,000617775	0,556587885	0,016458832	-0,0023222	0,819919376	0,311111512	0,092411387
9	CCL3	0,002234434	0,003972327	0,499836135	0,016588476	0,623140878	0,000621789	0,004581225	0,561720636	0,41092275	0,004545018
10	CXCL10	0,002635447	0,004216715	1,088263187	0,00404481	1,026171847	0,001694737	-0,001830658	0,89760759	0,143189011	0,576354422
11	IL18	0,003343045	0,004862611	0,29445298	0,043151471	0,427913827	0,000787352	0,008010002	0,149332607	0,362586745	0,000408353
12	CCL19	0,024739749	0,031668568	0,66784262	0,05451371	0,79600012	0,008216298	0,010810414	0,413481399	0,691829156	0,004306684
13	CCL23	0,025730712	0,031668568	0,233247324	0,142456659	0,373769993	0,006963569	0,010279354	0,092333457	0,19440581	0,077216893
14	IL-8	0,032968652	0,03767846	0,290171894	0,090757036	0,383785224	0,009965963	0,014852624	0,024687376	0,245472658	0,038817356
15	MCP-2	0,042023499	0,044825066	0,372599738	0,134993754	0,54201107	0,012309076	0,003930034	0,67940502	0,124032094	0,469124173
16	CCL4	0,060135795	0,060135795	-0,013477285	0,945524061	0,341452971	0,046223859	0,016006363	0,036228648	0,123709627	0,364429944

Supplementary Table 3: Comparison over time of protein concentrations after treated with AHSCT.

Linear mixed effects models adjusted for sex and age were used to estimate difference over time for the 16 proteins. Pairwise comparisons between time-points were estimated using the marginal means from the linear mixed linear models. The association with evidence of disease activity (EDA) was estimated within each time-point using the marginal means from the model.

	Protein	ANOVA (p-value)	ANOVA (q-value)	1 year vs 2 years (log2 fold change)	1 year vs 2 years (p-value)	1 year vs 5 years (log2 fold change)	1 year vs 5 years (p-value)	1 year vs baseline (log2 fold change)	1 year vs baseline (p-value)	2 year vs 5 years (log2 fold change)	2 year vs 5 years (p-value)	2 year vs baseline (log2 fold change)	2 year vs baseline (p-value)	5 year vs baseline (log2 fold change)
1	IL-12B	3,16E-09	3,40E-08	-0,165511592	0,393122434	0,048625652	0,892473734	-1,033181533	2,64E-08	0,214137244	0,552723575	-0,867669941	2,90E-05	-1,081807185
2	CCL3	4,24E-09	3,40E-08	0,088907718	0,249218987	0,351425007	0,022867977	-0,338762247	1,84E-06	0,262517289	0,081476834	-0,427669964	7,81E-07	-0,690187254
3	TNFRSF9	1,14E-06	6,09E-06	-0,04460359	0,616884295	0,034961611	0,83630044	-0,397191368	1,64E-06	0,079565202	0,637273737	-0,352587778	0,000212609	-0,432152979
4	sCD5	0,000113613	0,000454452	-0,168651763	0,197604274	-0,020952421	0,932039137	-0,503635128	2,45E-05	0,147699342	0,547550635	-0,334983365	0,013697692	-0,482682707
5	TNFSF14	0,000631903	0,00202209	-0,026388852	0,693389059	0,004409535	0,97299286	-0,227298134	0,000162102	0,030798387	0,810954863	-0,200909281	0,004783833	-0,231707668
6	CXCL9	0,001312426	0,003492381	0,053556933	0,685189334	0,360441514	0,154907958	-0,352981722	0,002630924	0,306884582	0,223115174	-0,406538655	0,00352805	-0,713423236
7	CXCL1	0,001527917	0,003492381	0,016323973	0,815857799	0,117606012	0,39592971	-0,207051709	0,000918949	0,101282039	0,456798363	-0,223375682	0,003067328	-0,324657721
8	CD8A	0,003247625	0,006495249	-0,049264486	0,616554856	-0,038399136	0,843871381	-0,304889077	0,000524392	0,01086535	0,954672658	-0,255624591	0,015314018	-0,26648994
9	CXCL10	0,016630285	0,029564951	-0,009132117	0,959080048	0,405972227	0,237467755	-0,402880346	0,010175879	0,415104344	0,223907668	-0,393748229	0,034631567	-0,808852573
10	CCL19	0,023467448	0,037547917	-0,126629772	0,40311072	0,118518567	0,685824799	-0,382100512	0,004254928	0,245148338	0,398828247	-0,25547074	0,106083148	-0,500619078
11	MCP-2	0,043006177	0,062554439	0,112460926	0,281956255	0,416654025	0,042078507	-0,110441947	0,220933046	0,304193099	0,13150574	-0,222902873	0,04221095	-0,527095972
12	IL-8	0,060741378	0,080988503	0,00930025	0,911476685	0,116340155	0,46830362	-0,165245239	0,024335711	0,107039905	0,502100606	-0,174545489	0,045530043	-0,281585394
13	CCL4	0,143440653	0,176542343	0,053656548	0,431155555	0,283912689	0,037327721	-0,031836269	0,587869608	0,23025614	0,084192725	-0,085492817	0,235666152	-0,315748957
14	IL18	0,230962286	0,263956898	0,087262143	0,111916284	0,147212928	0,179960497	-0,014278976	0,761655875	0,059950785	0,574107117	-0,101541119	0,0828629	-0,161491903
15	CCL23	0,330188013	0,352200547	0,064182169	0,26551841	0,17360768	0,128608423	-0,012193536	0,805604115	0,109425511	0,327678272	-0,076375705	0,20879842	-0,185801216
16	CXCL11	0,401711931	0,401711931	0,033646437	0,84958957	0,159517126	0,635035396	-0,211753734	0,170372279	0,125870689	0,70729237	-0,245400171	0,18018416	-0,37127086

	Protein	5 year vs baseline (p-value)	Baseline recidiv vs remission (log2 fold change)	Baseline recidiv vs remission (p-value)	1 year recidiv vs remission (log2 fold change)	1 year recidiv vs remission (p-value)	2 year recidiv vs remission (log2 fold change)	2 year recidiv vs remission (p-value)	5 year recidiv vs remission (log2 fold change)	5 year recidiv vs remission (p-value)	Age (log2 fold change)	Age (p-value)	Male vs Female (log2 fold change)	Male vs Female (p-value)
1	IL-12B	0,003666495	0,922031443	0,058381145	0,113427011	0,463636775	0,195113495	0,46518451	-0,667225953	0,192234429	-0,019368426	0,19284992	0,066314623	0,751205394
2	CCL3	2,75E-05	0,478003239	0,026329696	0,508875842	0,010043712	0,508778889	0,026375379	-0,017466442	0,966480736	0,008405723	0,478469286	0,5577037	0,000716346
3	TNFRSF9	0,013444933	0,523526553	0,015857807	0,14650464	0,242361731	0,170154813	0,315366745	0,061632878	0,905300168	-0,001793842	0,831320055	0,269009349	0,022466754
4	sCD5	0,055460018	0,643054127	0,047637108	0,21662553	0,164504846	0,28027716	0,200483122	-0,162724723	0,560735413	-0,010161543	0,374977122	0,038196486	0,811950854
5	TNFSF14	0,08532239	0,352409477	0,069871184	0,174608072	0,126433003	0,189657907	0,214383493	0,433384944	0,52457001	0,0036334	0,666721776	-0,144648055	0,218382856
6	CXCL9	0,006418217	0,726487199	0,023753253	0,242891898	0,294241585	0,137927395	0,533480347	0,215489843	0,773358494	0,022904	0,084122845	0,027306327	0,882887692
7	CXCL1	0,024738696	0,536103935	0,003758202	0,387801967	0,019532335	0,115997621	0,560422499	0,277948071	0,564196686	0,012245066	0,232282029	0,1141965	0,423091711
8	CD8A	0,188785443	0,316262451	0,309860744	0,222240093	0,316274548	0,149629067	0,579524474	1,725173165	0,156625756	0,013797069	0,362315321	0,256585546	0,22291059
9	CXCL10	0,022474796	0,854939717	0,048743196	0,322447536	0,325610917	0,567606809	0,106554074	1,216485974	0,338144352	0,020363912	0,297282244	0,212826268	0,435120664
10	CCL19	0,097438807	0,256087628	0,454758368	-0,171604202	0,595259616	0,183968871	0,662695489	-1,00210544	0,532749049	0,016536048	0,359272106	0,700375847	0,005344233
11	MCP-2	0,012641049	0,541845965	0,028982605	0,365162107	0,11572706	0,457993238	0,087962721	0,635690901	0,475388315	0,012714668	0,333695628	0,156819944	0,392002133
12	IL-8	0,087207813	0,45105671	0,016181606	0,129740341	0,390814645	0,123275095	0,502820536	0,338712611	0,602944296	0,014575426	0,094016842	0,340519327	0,005102718
13	CCL4	0,025487328	0,104111073	0,566727764	0,306917285	0,100623992	0,38157298	0,056823546	-0,066692977	0,846732481	0,009881243	0,34441707	0,193698202	0,182412675
14	IL18	0,158384102	0,170576277	0,245980871	0,049918588	0,788887472	0,034792319	0,85622279	-0,106297078	0,834842669	-0,003202345	0,732434567	0,225302983	0,083278598
15	CCL23	0,115906179	0,3231293	0,025506552	0,21124171	0,161594321	0,198769359	0,269551132	-0,208940101	0,735383324	0,007656407	0,37386493	0,120869782	0,312617658
16	CXCL11	0,278686037	0,355457275	0,36171103	0,432406329	0,150659039	0,517072859	0,099479953	0,929585811	0,433896087	0,003264075	0,840704268	-0,019123474	0,932991195

Supplementary Table 4: Comparison over time of protein concentrations after intrathecal rituximab treatment for PMS.

Linear mixed effects models adjusted for sex and age were used to estimate the difference over time for the 16 proteins. The association with disability progression was estimated within each time-point using the marginal means from the model.

	Protein	ANOVA (p-value)	ANOVA (q-value)	12 months vs 3 months (log2 fold change)	12 months vs 3 months (p-value)	12 months vs 6 months (log2 fold change)	12 months vs 6 months (p-value)	12 months vs Baseline (log2 fold change)	12 months vs Baseline (p-value)	3 months vs 6 months (log2 fold change)	3 months vs 6 months (p-value)	3 months vs Baseline (log2 fold change)	3 months vs Baseline (p-value)	6 months vs Baseline (log2 fold change)
1	CCL19	0,00475167	0,076026747	0,348666306	0,047731945	0,204620412	0,233478508	0,594434488	0,000881303	-0,144045894	0,407290106	0,245768182	0,159627566	0,389814077
2	CD8A	0,05110563	0,276709766	-0,052600129	0,672686517	-0,043896325	0,720420611	0,242920203	0,051065914	0,008703804	0,944236741	0,295520332	0,020178055	0,286816528
3	TNFRSF9	0,06323354	0,276709766	-0,129789658	0,096363883	-0,148660678	0,054106475	0,010591974	0,889228833	-0,018871021	0,806899757	0,140381632	0,072677455	0,159252653
4	MCP-2	0,06917744	0,276709766	-0,035865717	0,794164931	-0,007539525	0,955582902	0,278150633	0,043329723	0,028326192	0,83672521	0,314016351	0,02518974	0,285690158
5	CXCL1	0,27202048	0,650498153	-0,129834961	0,330768618	0,008050592	0,950998455	0,131753517	0,31658297	0,137885553	0,30192925	0,261588478	0,05275768	0,123702925
6	IL18	0,28738519	0,650498153	-0,109891647	0,097478275	-0,072674446	0,262938717	-0,012266231	0,849432875	0,037217201	0,570825835	0,097625416	0,140069467	0,060408215
7	CXCL10	0,31135861	0,650498153	0,058245008	0,705729892	0,163773198	0,283008103	0,264939415	0,084808367	0,105528189	0,494401161	0,206694407	0,183137297	0,101166217
8	CCL23	0,3297635	0,650498153	-0,049972804	0,452291667	0,011579396	0,859353165	0,071472528	0,276425087	0,0615522	0,355123669	0,121445332	0,070849131	0,059893132
9	CCL3	0,36590521	0,650498153	-0,098904853	0,227683058	-0,056797751	0,480085446	0,033547393	0,676360078	0,042107103	0,60566711	0,132452246	0,107734843	0,090345144
10	sCD5	0,49692447	0,715707722	0,052109261	0,493197507	0,066770688	0,373348313	0,113986826	0,130972892	0,014661427	0,846855415	0,061877565	0,416243151	0,047216138
11	TNFSF14	0,54238055	0,715707722	-0,04975601	0,47459325	-0,098169157	0,154617437	-0,064181452	0,349884297	-0,048413147	0,486558462	-0,014425442	0,835485249	0,033987705
12	CCL4	0,55212924	0,715707722	-0,016299242	0,854710999	-0,010996646	0,900175513	0,093689927	0,287501455	0,005302597	0,95248696	0,109989169	0,21935613	0,104686573
13	IL-8	0,58151252	0,715707722	-0,130215415	0,260012439	-0,076988312	0,497742616	0,006242553	0,956077259	0,053227103	0,643851271	0,136457968	0,238200065	0,083230866
14	CXCL9	0,65640182	0,750173514	-0,043338032	0,757588336	-0,168333702	0,226149318	-0,079962209	0,563611841	-0,12499567	0,37470549	-0,036624177	0,794222544	0,088371493
15	IL-12B	0,79138305	0,844141914	0,118540933	0,356175433	0,061209784	0,627731864	0,10183653	0,420680203	-0,057331149	0,654553009	-0,016704403	0,896207721	0,040626746
16	CXCL11	0,88449809	0,884498087	-0,011726231	0,926014474	0,02453254	0,843646988	0,080765962	0,516919293	0,036258771	0,774034275	0,092492193	0,464820798	0,056233422

	Protein	6 months vs Baseline (p-value)	Baseline progression vs stable (log2 fold change)	Baseline progression vs stable (p-value)	3 months progression vs stable (log2 fold change)	3 months progression vs stable (log2 fold change)	6 months progression vs stable (log2 fold change)	6 months progression vs stable (log2 fold change)	12 months progression vs stable (log2 fold change)	12 months progression vs stable (log2 fold change)	Age (log2 fold change)	Age (p-value)	Male vs Female (log2 fold change)	Male vs Female (p-value)
1	CCL19	0,02528568	0,943141789	0,144563183	0,923879726	0,103962491	0,700599016	0,125809469	0,358396506	0,351045715	0,022100314	0,323011371	0,347824084	0,347792239
2	CD8A	0,02200439	0,558432792	0,213499277	-0,036208875	0,918520551	-0,254890502	0,489297216	-0,432436966	0,205718213	-0,011123618	0,41662695	0,017541846	0,938260383
3	TNFRSF9	0,03948306	0,273324553	0,207358875	0,603854641	0,105127948	0,216884702	0,351809536	0,143019686	0,563533856	0,002661642	0,824395868	0,115772133	0,560941008
4	MCP-2	0,03805557	0,564023146	0,26757399	0,910759951	0,184481501	0,515765245	0,345879461	-0,04748029	0,923584287	0,035236517	0,12200426	-0,454217398	0,230127318
5	CXCL1	0,34668223	0,959559828	0,027289555	0,97004446	0,138887483	0,390949553	0,325201129	0,115788063	0,710714871	0,019495656	0,293064778	-0,696318951	0,023516265
6	IL18	0,35122475	0,295665617	0,188935734	0,189866109	0,552742213	0,002882068	0,991286118	-0,101441099	0,650557997	0,012459273	0,276164906	0,074146894	0,69660707
7	CXCL10	0,5058445	0,860474375	0,219356213	0,742006332	0,341532192	0,715033268	0,289651543	0,441229237	0,457617346	0,019853217	0,524112424	-0,615726576	0,235458371
8	CCL23	0,36083443	0,173199868	0,45414569	0,412125809	0,223565077	0,19959854	0,496904516	0,106749071	0,676418814	0,009701095	0,39653292	0,085841108	0,651686292
9	CCL3	0,2625267	0,260902122	0,585017098	0,663002842	0,191767001	0,346874006	0,473830975	0,092315553	0,831037249	-0,003933162	0,852253973	-0,203981101	0,563508128
10	sCD5	0,52826983	0,029558479	0,91664345	0,155018033	0,581644569	-0,026488127	0,932531361	0,037790272	0,873865777	-0,006745042	0,571021678	-0,289846723	0,142511263
11	TNFSF14	0,61954878	-0,246394029	0,338895887	0,391466945	0,169007687	0,203105137	0,444665108	0,030855902	0,896754541	0,003600486	0,739492933	-0,202097361	0,260850004
12	CCL4	0,23492287	-0,091222241	0,783981861	0,391427982	0,308955725	0,118232753	0,711341528	-0,194049551	0,620671567	-0,010144597	0,548043869	-0,016756342	0,952447469
13	IL-8	0,46364525	0,474465614	0,003262405	0,762427989	0,099948751	0,46767787	0,07489445	0,148804033	0,477156523	-0,000539044	0,96314019	-0,285743468	0,138149379
14	CXCL9	0,52329826	-0,25120822	0,577663361	0,159129748	0,778011582	-0,251695439	0,636493184	-0,036879198	0,931858326	0,039317149	0,072120932	-0,26091436	0,472183545
15	IL-12B	0,74736667	0,673596582	0,030016603	0,998506914	0,079723885	0,770858401	0,103133552	0,383877876	0,35172517	-0,016008413	0,478989941	-0,335866012	0,371600713
16	CXCL11	0,65129571	0,497729709	0,373377085	0,799006478	0,251501787	0,576935795	0,354560737	0,289622397	0,553124126	0,026204706	0,331081038	-0,846775514	0,059486569
